# Nuclear compartmentalization at the G1/S transition plays a key role in DNA replication control

**DOI:** 10.1038/s41467-026-75264-6

**Published:** 2026-08-10

**Authors:** Asami Oji, Kosuke Yusa, Izumi Noda, Takako Ichinose, Yoshiko Kondo, Ichiro Hiratani

**Affiliations:** 1 Laboratory for Developmental Epigenetics, RIKEN Center for Biosystems Dynamics Research (BDR), Kobe, Japan; 2 Laboratory of Stem Cell Genetics, Institute for Life and Medical Sciences, Kyoto University, Kyoto, Japan; 3 Present Address: Laboratory of Pathology and Development, Institute for Quantitative Biosciences, The University of Tokyo, Tokyo, Japan

**Keywords:** Chromosomes, Chromatin structure, Nuclear organization, DNA replication

## Abstract

Upon entry into the G1 phase following mitosis, mammalian chromosomal DNA becomes spatially segregated into A and B compartments, which correspond closely to classic euchromatin and heterochromatin, respectively. The functional significance of this spatial segregation, however, has remained unexplored due to the lack of means to manipulate this level of chromosomal organization. Through a genome-wide loss-of-function CRISPR screen, we identify GINS4, a component of the replicative DNA helicase complex, as a factor essential for segregation of A and B compartments during the G1-to-S phase transition. Using GINS4 depletion experiments, we show that proper A/B compartment organization at the time of S-phase entry plays a key role in efficient DNA synthesis. Furthermore, DNA synthesis with attenuated A/B compartments is associated with defects in replication timing regulation. Our findings uncover a previously unrecognized role for GINS4 in regulating nuclear architecture and underscore the biological significance of nuclear compartmentalization in DNA replication control.

## Abstract (web summary)

The genome is organized into nuclear compartments that correlate with replication timing. Here, the authors show that GINS4 is required for compartment organization at the G1/S transition, which supports DNA replication control, revealing a key role for nuclear architecture in genome duplication.

## Introduction

Faithful replication of DNA is essential for genome integrity. To achieve this, eukaryotic cells temporally separate the processes of replication origin licensing (in G1-phase) and initiation (in S-phase), thereby avoiding re-replication^1–3^. According to the current paradigm, licensing involves a multi-step assembly of the pre-replicative complex (pre-RC) in G1-phase at each potential origin of replication on the genome that eventually forms the minichromosome maintenance protein 2–7 (MCM2–7) double hexamer^1–3^. Upon transition into S-phase, a subset of the MCM2–7 double hexamers are transformed into active replicative helicases, the CMG (CDC45-MCM2–7-GINS) complexes, which travel with the replication fork to unwind and synthesize DNA^1–3^.

Furthermore, eukaryotes, particularly multi-cellular organisms, subdivide their large genomes into early or late replication domains to execute the genome duplication process in a “divide and conquer” manner^4–6^. Early and late replication timing (RT) correlates well with euchromatic A and heterochromatic B compartments in the interphase nucleus, respectively, identified by high-throughput chromosome conformation capture (Hi-C)^7^. This RT program is likely imposed by the distinct chromatin structures in different parts of the chromosomes that translate to distinct chromatin accessibility of rate-limiting replication factors involved in active helicase formation^2^. For instance, RIF1 localizes to late-replicating chromatin and prevents it from replicating early through its ability to suppress MCM phosphorylation and helicase activation^8^. Interestingly, once the RT is disrupted and randomized genome-wide by RIF1 depletion, cells fail to maintain their epigenome, suggesting the importance of RT in maintaining the global epigenetic state^9^.

It is known that A/B compartments disappear during mitosis but are re-established in early G1 following cell division^10–19^. Interestingly, the timing of A/B compartment re-establishment is coincident with the establishment of the early/late RT program^14^, which raises the possibility of A/B compartments being involved in RT regulation. For instance, RIF1 is also involved in the regulation of chromatin organization in G1-phase, suggesting this possibility^20^. However, such a possibility has never been tested due to our inability to manipulate A/B compartments and directly assess their impact on RT.

To fill this gap, we devised a GFP-based RT reporter system and used it to perform a genome-wide screening for RT regulators in mouse embryonic stem cells (mESCs). We aimed to obtain a list of RT regulators and use it to further identify previously unrecognized regulators of A/B compartments through a secondary validation using Hi-C. Among the top hits from the primary screening were the GINS (Go-Ichi-Nii-San; five, one, two, and three in Japanese; Sld5-Psf1-Psf2-Psf3) complex subunits, which are essential components of the replication machinery^21^. A detailed analysis of the GINS4 subunit function revealed its unexpected role in the regulation of nuclear compartments independent of DNA synthesis. Moreover, it enabled experimental manipulation of A/B compartment strength and assessment of its importance in replication and RT control.

## Results

### Development of a GFP reporter system for RT regulator screening

**Fig. 1:**
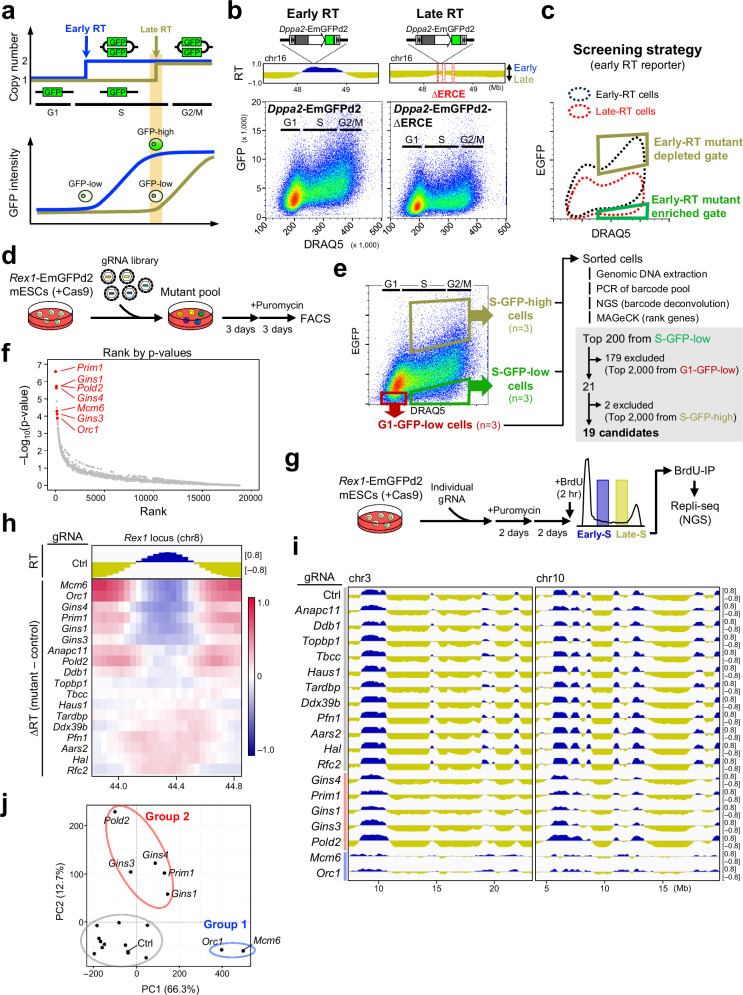
Genome-wide loss-of-function screening using a GFP-based RT reporter identified several candidate RT regulators in mESCs. **a** Schematic diagram of GFP copy number and fluorescence intensity kinetics during the cell cycle. **b***Dppa2* locus RT profiles (top) and FACS profiles (bottom) of ERCE-intact and -deleted cells. EmGFPd2 fragment was inserted into the early-replicating *Dppa2* locus in the parental clone. *X*- and *Y*-axes indicate DRAQ5 (DNA dye) and GFP intensity, respectively. **c** A FACS gating strategy to screen late-RT mutants from cells with a GFP reporter in an early-replicating locus based on the FACS profiles of (**b**). **d** Schematic of mutant pool preparation using a genome-wide lentiviral gRNA library. Lentiviral-infected cells are selected by puromycin treatment. **e** Left: a FACS plot of mutant pool generated from the *Rex1*-EmGFPd2 cell line (early-RT). Sorting gates are shown as colored boxes. Three independent experiments were performed. Right: each process after sorting. The gray highlight describes how the candidate genes were filtered. **f** Screening results by MAGeCK^31^, using three independent replicates. Genes were ranked by nominal gene-level P values calculated with MAGeCK’s modified robust rank aggregation (α-RRA) test, based on gRNA ranks from one-sided negative-binomial tests for positive selection. The y-axis shows −log10(nominal P value). High-ranked DNA replication factors are shown in red. **g** Scheme of the bulk-KO method to individually validate candidate genes. Individual gRNA-transfected cells were selected by puromycin treatment and sorted with early- and late-S gates, and BrdU-substituted DNA was immunoprecipitated with BrdU antibody, followed by Repli-seq. Two different gRNAs were tested per candidate gene. **h** The RT profile around the *Rex1* locus in control cells is at the top (200-kb bins), while the heatmaps show ∆RT (mutant–control) of each bulk-KO mutant. Mutants were sorted in ascending order of the ∆RT value at the center bin of the *Rex1* locus. Bulk-KO RT values of two gRNAs were averaged. **i** RT profiles of bulk-KO mutants (Chromosomes 3 and 10). Each row shows an averaged RT profile obtained from two gRNAs. **j** PCA plots generated from RT profiles of each mutant, showing three distinct groups.

To identify RT regulators by a high-throughput CRISPR screening, we thought to visualize RT using flow cytometry. We inserted a CAG-GFP cassette into a target genomic region of known RT (early or late S) in mESCs and monitored its copy number by GFP intensity. Since the CAG promoter^22^ increases gene expression by two-fold following replication^23^, we reasoned that we could distinguish the replication state (i.e., copy number) of the GFP-inserted locus based on the GFP intensity in S-phase. Cells that have replicated GFP in early S-phase should theoretically exhibit twice as high GFP expression toward the end of S-phase than cells that have replicated GFP in late S-phase (Fig. 1a).

To ensure that the GFP intensity of a given cell-cycle phase was not affected by any leftover GFP from the previous phase, we constructed a destabilized form of GFP with a half-life of 2 h (GFPd2)^24^, which was further modified to improve brightness (Emerald GFPd2 or EmGFPd2)^25^ (Supplementary Fig. 1a, b). The CAG-EmGFPd2 sequence was flanked by the 3′D4Z4 and HS4 insulator elements, which reportedly block silencing by local repressive histone modifications and DNA methylation^26^. This GFP cassette was inserted into the following gene neighborhoods with characteristic RT in mESCs: *Zfp42/Rex1* (*Rex1*-EmGFPd2; early RT), *Dppa2* (*Dppa2*-EmGFPd2; early RT), and *Olfr596* (*Olfr596*-EmGFPd2; late RT) (Supplementary Fig. 1c, d). Despite the relatively high cell-to-cell variation in GFP intensity, *Rex1*- and *Dppa2*-EmGFPd2 mESC lines clearly showed a gradual GFP increase toward the end of S-phase, while the *Olfr596*-EmGFPd2 line only showed a mild increase in GFP intensity as hypothesized in (Fig. 1a and Supplementary Fig. 1e, f). Importantly, the GFP cassette insertion was homozygous in the *Rex1*-EmGFPd2 cell line, resulting in higher GFP expression compared to the *Dppa2*- and *Olfr596*-EmGFPd2 cell lines with heterozygous knock-ins (Supplementary Fig. 1d–f). These results suggest that RT of the EmGFPd2-inserted locus could be estimated from GFP expression levels in S-phase by flow cytometry.

While the S-phase GFP kinetics in the EmGFPd2 mESC lines were consistent with our hypothesis, an ultimate proof-of-concept experiment is to demonstrate that changing the RT of a specific reporter locus results in altered GFP kinetics. We took advantage of the early replication control elements (ERCEs), which are cis-acting elements necessary for maintaining the early RT of the *Dppa2* replication domain^27^. We performed Repli-seq^28^ to measure RT genome-wide and confirmed that ERCE deletion in the *Dppa2*-EmGFPd2 mESCs indeed caused the *Dppa2* replication domain to replicate in late S-phase (Fig. 1b and Supplementary Fig. 1g), which allowed us to directly compare the effect of RT differences on the expression kinetics of the *Dppa2*-EmGFPd2 reporter. In contrast to *Dppa2*-EmGFPd2 mESCs with intact ERCEs, the ERCE-deletion mutant showed a relatively constant GFP expression throughout the cell cycle, as in the *Olfr596*-EmGFPd2 cell line (Fig. 1b and Supplementary Fig. 1h). Thus, an RT change of a reporter locus indeed translated into changes in GFP kinetics, particularly in late S-phase, leading us to conclude that our reporter system should work as anticipated for comprehensive screening of RT regulators.

### A genome-scale screen identified replication factors as RT regulators

We decided to use homozygous *Rex1*-EmGFPd2 mESCs, which expressed two-fold higher GFP than heterozygous cells (Supplementary Fig. 1d, f). A gating strategy to identify genes required for early replication of the *Rex1* locus was simulated (Fig. 1c) based on the flow cytometry profile of ERCE-deleted *Dppa2*-EmGFPd2 mESCs (Fig. 1b). In brief, we aimed to identify mutants that replicated the *Rex1* locus late and thus exhibited low GFP expression toward late S-phase like the ERCE-deletion mutant (Fig. 1c). We then inserted an EF1a-Cas9 transgene into the safe-harbor *Rosa26* locus and selected a clone (#11) with the strongest CAS9 activity by evaluating the degree of GFP reduction by EmGFPd2-targeting gRNAs (Supplementary Fig. 2a, b). Using this clone, we set out to perform a genome-scale CRISPR-knockout (KO) screen (See “Methods”)^29^. Cells were transduced with a lentiviral library composed of 112,058 gRNAs targeting 18,805 mouse genes^30^ at a low multiplicity of infection (~0.3) to achieve single gRNA integration per cell (Fig. 1d and Supplementary Fig. 2c). After puromycin selection to eliminate untransduced cells, we harvested the remaining cells for fluorescence-activated cell sorting (FACS). We sorted the GFP-low population in late S-phase to identify enriched mutants (Fig. 1e, S-GFP-low). One legitimate concern was the false discovery of genes that affected GFP intensity without changing RT, such as genes involved in protein stability regulation. Because such effect would not be limited to late S-phase but observed throughout the cell cycle, we sorted the GFP-low population in G1-phase to filter out genes enriched in this population (Fig. 1e, G1-GFP-low). We also sorted the GFP-high population during late S-phase to remove genes enriched in this population (Fig. 1e, S-GFP-high).

After sorting ~3 × 10^6^ cells per FACS gate in three independent experiments, gRNA sequences integrated into their genome were amplified by PCR and read by next-generation sequencing (NGS), followed by statistical analysis using the MAGeCK algorithm^31^ (Fig. 1e). To identify genes required for early replication of the *Rex1* locus, we started with a list of the top 200 genes enriched in the S-GFP-low gate, excluded the top 2000 G1-GFP-low genes from the list, and further excluded the top 2000 S-GFP-high genes. As a result, we obtained a list of 19 candidates (Fig. 1e, f and Supplementary Fig. 2d). Gene Ontology (GO) analysis by Gene Set Enrichment Analysis (GSEA)^32^ revealed significant enrichment of DNA replication-related genes (Supplementary Fig. 2e), indicating enrichment of reasonable candidates by unbiased genome-wide screening.

To validate the phenotypes of the 19 top hits, we performed Repli-seq of mESCs transfected with the individual gRNAs used in the screen (Fig. 1g and Supplementary Fig. 2f). Control cells were transfected with empty vectors lacking gRNAs. By this bulk-knockout method (bulk-knockout generates a mixed population of various indels; see Methods), 10 out of 19 mutants showed RT delay (∆RT < 0) at the *Rex1* locus, consistent with the screening results (Fig. 1h, ∆RT shows RT differential between mutant and control; Supplementary Fig. 2g). RT profiles varied among the mutants, and three clusters were identified by principal component analysis (PCA) (Fig. 1i, j). Mutants that clustered together with control did not show much RT change, and the remaining two clusters were termed groups 1 and 2. The group-1 factors (*Orc1* and *Mcm6*) were pre-RC components that are loaded onto chromatin earlier than the group-2 factors (*Prim1*, *Gins1*, *Gins3*, *Gins4*, and *Pold2*), which are recruited to the replisome (replication machinery) just before replication initiation. The clear difference in the RT profiles of group-1 and group-2 mutants (Fig. 1i) indicates that RT defect severity correlates with the order of events during replisome assembly.

As replisomes are essential for DNA replication, it was puzzling that such mutants could continue DNA synthesis and appear in our screening. Since our CRISPR screen can only identify viable mutants, we assumed them to be hypomorphic (partial loss of gene function). To confirm this, we employed the auxin-inducible degron 2 (AID2) protein knockdown (KD) technology^33^. Since *Mcm6* and *Orc1* mutants showed drastic RT defects genome-wide and *Gins4* mutant showed the largest RT delay of the *Rex1* locus within group-2 (Fig. 1h and Supplementary Fig. 2g), we chose them to represent RT profile groups 1 and 2, respectively, and introduced the mAID tag biallelically for further analysis (Supplementary Fig. 3a–d).

**Fig. 2:**
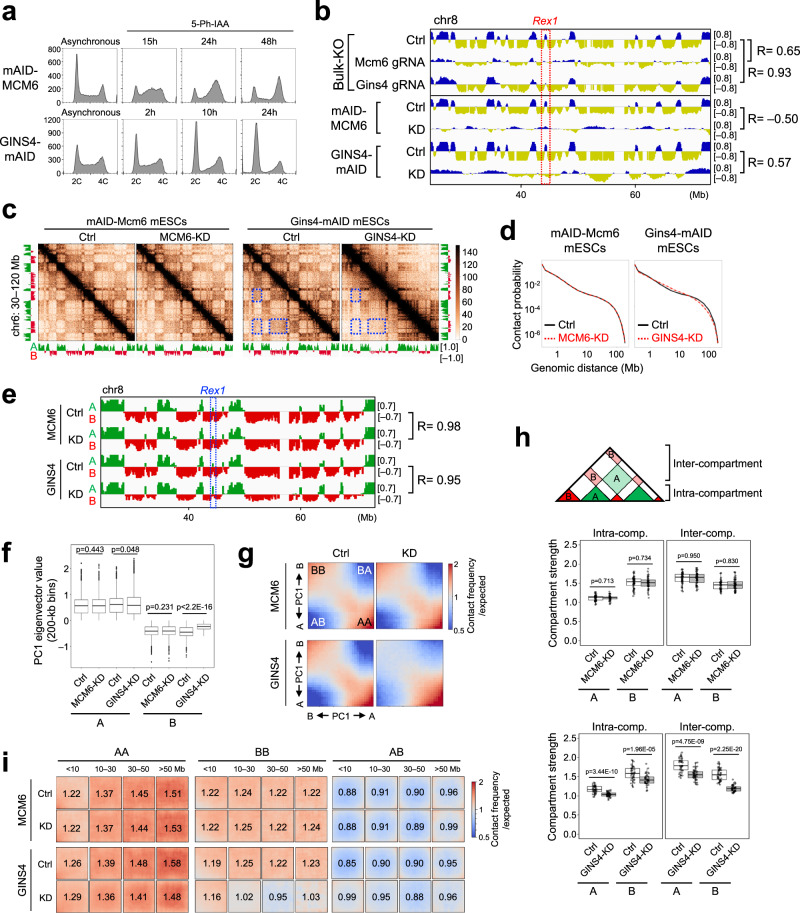
Auxin-mediated GINS4 depletion affected not only RT but also the 3D genome architecture. **a** Time-course cell-cycle profiles of Auxin-mediated MCM6- and GINS4-KD cells after adding 5-Ph-IAA. Treatment duration was adjusted to observe cell cycle progression in a step-wise manner. Cells were stained with Propidium iodide. **b** Comparison of RT profiles between bulk-KO and auxin-mediated KD. Severe RT phenotypes were observed by MCM6- and GINS4-depletion for 24 h. Red dotted box indicates the position of the early replicating *Rex1* locus. Each row shows an averaged RT profile obtained from two gRNAs (bulk-KO) and two replicates (auxin-mediated KD). Pearson’s correlation coefficient values are shown on the right. **c** Hi-C contact heatmaps of auxin-mediated MCM6- and GINS4-depleted cells (5-Ph-IAA treatment for 24 h) and control cells (no 5-Ph-IAA treatment). PC1 eigenvector values (A/B compartments) are calculated and shown beside and below the Hi-C contact heatmaps. Blue dotted boxes indicate contacts between B-compartment regions, which are markedly decreased in GINS4-depleted cells. Two independent experiments showed similar results. **d** Relative contact probability plots generated from Hi-C data of MCM6- and GINS4-KD cells (red dotted lines) and control cells (black solid lines). **e** PC1 eigenvector values calculated from Hi-C data (200-kb bins) for MCM6- and GINS4-KD cells. Blue dotted box indicates the *Rex1* locus position. Pearson’s correlation coefficient values are shown on the right. (**f**) Distribution of PC1 eigenvector values (200-kb bins) genome-wide. Two biological replicates were merged for each condition. *P* values were calculated using two-sided Wilcoxon rank-sum tests between control and KD samples. For box plots, the center line indicates the median, box bounds indicate the first and third quartiles, whiskers extend to the most extreme values within 1.5× the interquartile range, and points indicate outliers. **g** Saddle plots of Hi-C data for MCM6- and GINS4-depleted cells. Saddle plots were generated by sorting the genomic bins in ascending order of the Hi-C PC1 scores. **h** Top, schematic diagram of intra- and inter-compartment contacts. Bottom, intra- and inter-compartment interaction strengths calculated for A or B compartments in MCM6- and GINS4-depleted cells using the Pentad tool^38^. Two biological replicates were merged for each condition. The mean value of contacts within A and B compartments is divided by the mean value of contacts between A and B compartments. Each dot represents a single chromosome. *P* values were calculated using two-sided Wilcoxon rank-sum tests between control and KD samples. For box plots, the center line indicates the median, box bounds indicate the first and third quartiles, whiskers extend to the most extreme values within 1.5× the interquartile range. (**i**) Distance-dependent contact frequency of MCM6- and GINS4-depleted cells analyzed by Pentad. Numbers at the center of the plots indicate the average value of contact frequency/expected. Source data for (**f**, **h**) are provided as a Source data file.

While ORC1-KD did not reproduce the RT defects observed in bulk-KO cells (Supplementary Fig. 3e–g), MCM6-KD and GINS4-KD caused more widespread RT defects than bulk KO and subsequent cell cycle arrest, supporting the idea that bulk-KO phenotypes of *Mcm6* and *Gins4* were indeed caused by hypomorphic alleles (Fig. 2a, b, Supplementary Fig. 2f and Supplementary Fig. 3h, i). The ORC1-KD phenotype was consistent with a recent report demonstrating that ORC1 depletion by AID2 does not inhibit DNA replication^34^. Despite widespread RT defects, both MCM6- and GINS4-KD cells continued to slowly synthesize DNA, presumably using residual proteins and/or partial CMG complexes to complete genome replication in S-phase. Eventually, however, the cells were arrested at G2/M-phase (MCM6-KD) or G1-phase (GINS4-KD) (Fig. 2a), possibly by checkpoint activation. For instance, reduction of MCM levels is known to induce a checkpoint response that prevents mitotic division^35^.

### GINS4 but not MCM6 is required for 3D genome organization

As we successfully generated two types of RT-defective mutants, we next performed Hi-C to investigate whether MCM6-KD and GINS4-KD cells show any A/B compartment defects. Consistent with a previous report in mouse B cells^36^, MCM6-KD cells exhibited a normal Hi-C interaction profile despite their widespread RT defects (Fig. 2c and Supplementary Fig. 4a). This indicates that, under certain circumstances, RT defects do not translate to A/B compartment defects and the two can be uncoupled. By contrast, GINS4-KD Hi-C data were visibly different from the control (Fig. 2c and Supplementary Fig. 4a), showing an increase in short-range intra-chromosomal interactions (<10 Mb) and a loss of long-range interactions (>10 Mb) (Fig. 2c, d and Supplementary Fig. 4a). As a result, GINS4-KD formed an independent cluster when the Hi-C data were compared using stratum-adjusted correlation coefficient (Supplementary Fig. 4b)^37^. Although the overall A/B compartmentalization pattern (Hi-C principal component 1, PC1) including the *Rex1* locus looked unaffected, absolute PC1 values of B-compartment domains in GINS4-KD cells were significantly smaller than control cells (Fig. 2e, f). We plotted interactions between loci arranged by their PC1 values (i.e., saddle plots), which revealed markedly decreased interactions between B-compartment domains (B-B interactions) and increased A-B interactions by GINS4-KD but not MCM6-KD (Fig. 2g).

To further examine the chromatin interaction patterns, we utilized the Pentad tool^38^ to calculate the average short- and long-range compartment interaction strength. Pentad allows the analysis of compartment strength within and between each compartment by dividing the mean A-A and B-B contacts by the mean A-B contact values. We observed an overall decrease in both intra- and inter-compartment scales upon GINS4-KD but not MCM6-KD (Fig. 2h), presumably due to the increase of A-B contacts in the former. The stratified compartments by genomic distance exhibited decreased long-range B-B interactions (>10 Mb) and increased short-range A-B interactions (<10 Mb) in GINS4-KD but not MCM6-KD (Fig. 2i), which can be observed in the Hi-C contact maps (Fig. 2c and Supplementary Fig. 4a). These results suggest that GINS4 is required, either directly or indirectly, for long-range B-B interactions and A/B compartment segregation. Although both GINS4 and MCM6 are components of the CMG complex and affect replication upon KD, only GINS4 depletion affected nuclear compartmentalization, underscoring the specificity of GINS4 in compartment regulation.

### GINS4 is required for maintenance/maturation of B-compartment strength

**Fig. 3:**
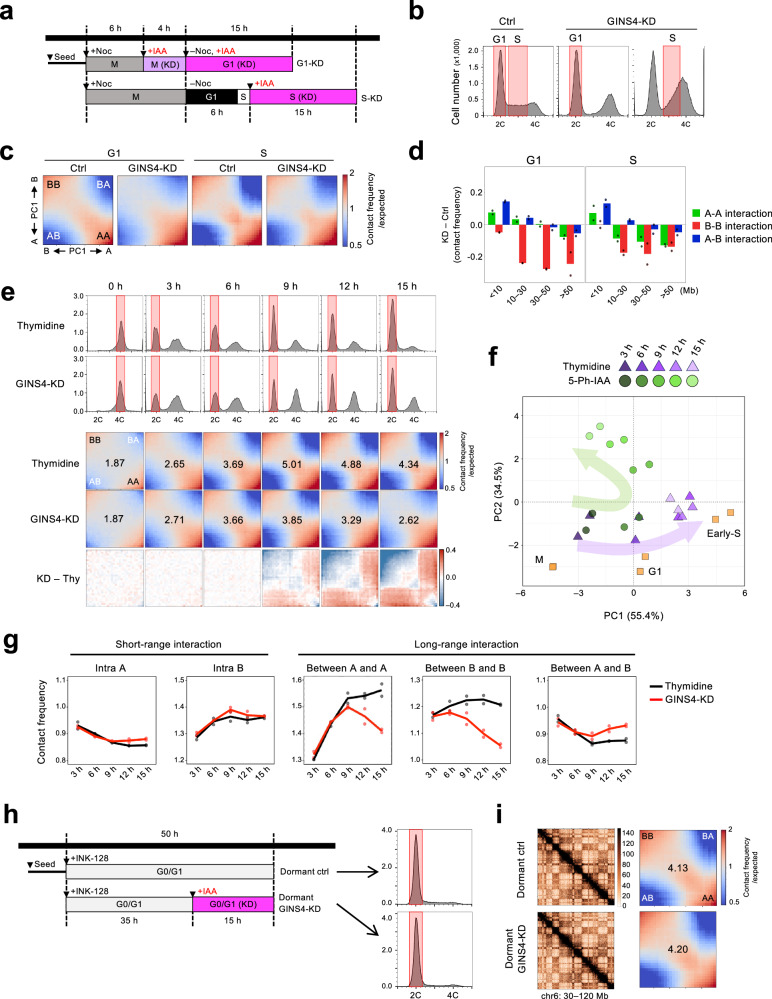
GINS4 is required for maintenance/maturation of long-range interaction between B-compartment domains during the G1-to-S phase transition. **a** Scheme of cell cycle synchronization to obtain G1- or S-phase cells depleted of GINS4. Noc, nocodazole; IAA, 5-Ph-IAA. **b** FACS plots of asynchronous control cells (Ctrl) for normal G1- and S-phase population sorting and 5-Ph-IAA-treated cells (GINS4-KD) with cell-cycle synchronization described in (**a**). Cell populations highlighted in red were sorted. Cells were stained with Propidium iodide. **c** Saddle plots of Hi-C data for G1- and S-phase cells with or without GINS4 depletion. Two independent experiments showed similar results. **d** Differences in the A-A, B-B, and A-B contact frequency between control and GINS4-KD (GINS4-KD–Ctrl). Subtraction values are the average of two replicates. Black dots indicate individual values of each replicate. **e** Top panel, FACS plots of prometaphase-arrested cells (0 h) and cells released from prometaphase arrest (3–15 h) in the presence of thymidine (Thymidine) or 5-Ph-IAA (GINS4-KD). Cell populations highlighted in red were sorted for Hi-C. Cells were stained with Propidium iodide. Bottom panel, saddle plots generated from Hi-C data and subtracted heatmaps (KD–Thy). Numbers at the center of the heatmaps indicate compartment strength calculated as a ratio of (AA + BB)/(AB + BA) using the median values of top 20% of A-A, B-B, and A-B interactions. **f** PCA plots generated from saddle plot profiles of time-course Hi-C samples shown in (**e**) (3–15 h samples). M (metaphase) samples correspond to nocodazole-treated cells shown as 0 h in (**e**). Hi-C data from G1 and early-S samples were sorted based on the gates in Supplementary Fig. 5b. **g** Plots of chromatin contact frequency dynamics calculated by Pentad. Contact frequency within the same compartment is shown as short-range interactions (left), while those between compartments are shown as long-range interactions (right). Black dots, thymidine-treated samples; red dots, GINS4-KD samples; solid lines, the average of two replicates. **h** Scheme of G0/G1 arrest (‘dormant’ state) induction in mESCs with or without 5-Ph-IAA treatment. Right panels are the FACS plots of cells after each treatment. **i** Hi-C contact heatmaps and saddle plots of INK-128-treated (“dormant”) cells, with or without 5-Ph-IAA treatment. Numbers at the center of the heatmaps indicate the compartment strength. Source data for panels (**d**, **g**) are provided as a Source data file.

Because most of the 5-Ph-IAA-treated GINS4-KD cells eventually reached G1-phase (Fig. 2a), it was unclear when the defects in compartment organization emerged during GINS4 depletion by 5-Ph-IAA treatment. To address this, we induced GINS4 depletion in a G1- or S-phase-specific manner and compared their A/B compartment integrity with untreated G1- and S-phase cells (Fig. 3a, b). To achieve G1-KD of GINS4, cells were first subjected to mitotic arrest with nocodazole, followed by 5-Ph-IAA treatment to deplete GINS4 before cell division. These cells were then released from nocodazole but still treated with 5-Ph-IAA for additional 15 h, resulting in G1 arrest with GINS4 depletion. G1 population was purified by FACS to avoid contamination of cells from other cell-cycle phases (Fig. 3b and Supplementary Fig. 4c). To achieve S-KD of GINS4, cells were treated with 5-Ph-IAA for 15 h once the synchronized cells entered S phase, and cells in S-phase were collected by FACS. We also sorted G1- and S-phase fractions from untreated asynchronous cells as control samples (Fig. 3b).

Cell-cycle-phase-specific Hi-C of control samples revealed stronger compartment segregation in S-phase than in G1-phase, as shown by the clearer checkerboard pattern in the Hi-C heatmap (Supplementary Fig. 4d) and decrease of A-B interaction in S-phase cells (Fig. 3c), consistent with a previous report^13^. By comparing the saddle plots, we found that G1-KD cells exhibited marked B-B interaction loss, while S-KD cells showed an overall decrease in A-A and B-B contact frequency (Fig. 3c). Pentad analysis quantitatively revealed that both GINS4 G1- and S-KD exhibited the same tendency of compartment aberrancy, i.e., decreased long-range A-A and B-B interactions and increased short-range A-B interactions (<10 Mb) (Supplementary Fig. 4e). Notably, the degree of B-compartment weakening was more pronounced in G1-KD than in S-KD (Fig. 3d). From these results, we concluded that GINS4 contributes to compartment integrity not only during S-phase, but also before replication initiation.

As previous reports have shown that A/B compartments are re-established during early G1-phase^14–16,18^, we next asked whether the drastic B-B interaction loss observed in GINS4 G1-KD cells was due to the failure of compartment re-establishment after cell division or the maintenance/maturation of the re-established compartments. To address this, we arrested cells with nocodazole, depleted GINS4, and then performed time-course Hi-C at 3 h intervals after release from the nocodazole block (Supplementary Fig. 5a). Because GINS4-KD cells accumulated at the 2C DNA content peak (Fig. 2a), we reasoned that it would be preferable to have the control sample in a non-replicating state after cell division. To achieve this, we treated cells with thymidine following release from nocodazole to arrest them at the G1/S boundary. In addition, to confirm that the 3D genome organization of the thymidine-treated cells properly progressed from metaphase to G1/S after release from nocodazole, we sorted G1 and early S-phase cells from asynchronous population and performed Hi-C for comparison (Supplementary Fig. 5b).

Consistent with previous reports^11,12^, metaphase cells exhibited a lack of compartment segregation (Fig. 3e and Supplementary Fig. 5c, 0 h). During the first 9 h after release from nocodazole, thymidine-treated cells showed gradual A and B compartment segregation, which became enhanced over time (Fig. 3e and Supplementary Fig. 5c). The saddle plot profiles generated from 3, 6, and 9 h thymidine-treated cells clearly formed a trajectory of cell cycle progression, aligning well with G1 and early-S samples, respectively (Fig. 3f). Thereafter, the 12- and 15 h thymidine-treated samples remained positioned close to the 9 h thymidine and early-S samples on the PCA plot, suggesting that thymidine-treated cells properly reorganize their compartments in a manner similar to how it occurs during cell cycle progression. Thus, we reasoned that thymidine-treated cells could serve as a suitable control for GINS4 G1-KD cells (see Supplementary Note 1 for more details on the rationale of control selection).

By contrast, although GINS4-depleted cells established A and B compartments during the first 6 h in a manner similar to thymidine-treated control cells, they started to exhibit gradual increase in A-B interactions and decrease in B-B interactions after the 9 h time point (Fig. 3e and Supplementary Fig. 5c), forming a distinct trajectory on the PCA plot (Fig. 3f). The higher 4C peaks in GINS4-KD cells, particularly at the later time points (Fig. 3e, 9–15 h), likely reflect the incomplete nature of nocodazole-induced prometaphase arrest. That is, because 5-Ph-IAA was added 4 h prior to nocodazole removal (Supplementary Fig. 5a) to ensure complete degradation of GINS4 before G1 entry, a small subset of cells that remained in late S phase at 0 h may have proceeded through the cell cycle very slowly under conditions of GINS4 depletion, thereby becoming a source of 4C cells at later time points in the GINS4-KD cells but not thymidine control cells. Interestingly, when we calculated the contact frequency for each compartment type, a decrease in long-range inter-B compartment strength was the earliest sign of a compartment-level chromatin contact change in GINS4-KD cells (Fig. 3g), suggesting that compartment defects in GINS4-KD initiated from the distal B-B interactions and subsequently influenced other types of chromosome interactions. We conclude that GINS4 functions during the maintenance and/or maturation but not the initiation phase of A/B compartment establishment, with its primary target being the B-B interactions.

### GINS4 acts to regulate the A/B compartments during the G1-to-S transition

While our data suggest that GINS4 plays a role in maintenance and/or maturation of the A/B compartments at some point between the G1 and S phases, it remains unclear whether it acts during G1 or at the G1/S boundary, especially given that GINS4 is a replicative factor with well-known function in S-phase. To get a handle on this problem, we examined the S-phase marker, Geminin. After arresting and releasing cells with nocodazole, followed by overnight treatment with either thymidine or 5-Ph-IAA, we observed that the cell-cycle-arrested cells with a 2C DNA content were Geminin-positive in both conditions (Supplementary Fig. 6a). This indicates that these cells were arrested at the G1/S boundary; more precisely, immediately after entering S-phase. This observation prompted us to explore drugs that enabled the investigation of GINS4 function in truly G1-arrested cells, which should be Geminin-negative.

We tested several compounds reported to induce G1 arrest (Supplementary Fig. 6b)^9,39–41^ and identified INK-128, an mTOR inhibitor, as an excellent candidate. When tested, 1 µM INK-128 reversibly arrested mESCs in G0/G1 (Supplementary Fig. 6c), and proliferation markers Ki-67 and Geminin were both negative and hence, dormant (Supplementary Fig. 6d, e). Surprisingly, upon GINS4-KD in G0/G1-arrested “dormant” mESCs using INK-128 followed by Hi-C, we saw no effect on the B-B interactions and the overall 3D genome organization (Fig. 3h, i). Cells exhibited a normal checkerboard pattern and a G1-like saddle plot profile (Fig. 3i and Supplementary Fig. 6f). These results suggest that GINS4 functions in compartment regulation during the G1-to-S transition but not G1. Our time-course Hi-C data showed that the loss of B-B interactions occurred within 3–6 h after release from nocodazole (Fig. 3g). As the G1-phase of mESCs under our culture conditions is ~3 h^42^, this observation is also consistent with the idea that GINS4 plays a role in 3D genome regulation during the G1-to-S transition.

The prevailing view holds that A/B compartments are rapidly established as cells exit mitosis and enter G1 phase^10,14–18^. That GINS4 is involved in compartment regulation during the G1-to-S transition appears to contradict this view. However, whether the establishment of A/B compartments is fully completed within the G1 phase remains an open question, as these studies did not analyze A/B compartment behavior across the entire cell cycle. Moreover, a single-cell Hi-C study by Nagano et al. ^13^ reported that A/B compartment strength is relatively weak in G1 and continues to increase during the S and G2 phases, albeit at modest resolution. Consistent with this, our Hi-C analysis revealed that S-phase cells exhibit a markedly higher degree of compartment segregation than G1 cells (Supplementary Fig. 5b). Thus, our findings regarding the role of GINS4 in 3D genome regulation during the G1-to-S transition suggest that A/B compartment reorganization is not completed in G1 but instead continues as cells enter S-phase, warranting a reevaluation of the current view in the field.

Importantly, the G1-to-S transition is the time at which GINS4 is loaded onto the genome as part of the CMG helicase for replication initiation. In an attempt to investigate whether GINS4 functions in 3D genome regulation as part of the GINS complex, we decided to analyze the function of GINS1, GINS2, and GINS3. To this end, we individually tagged them with AID and depleted each protein by 5-Ph-IAA treatment (Supplementary Fig. 7a–c). Unexpectedly, depletion of GINS1, GINS2, or GINS3 did not result in an accumulation of cells at the G1/S boundary (Supplementary Fig. 7d). Although these cells exhibited mild replication defects, they continued to proliferate, unlike the rapid cell death observed in GINS4-KD cells (Supplementary Fig. 7d).

By western blotting, we found that depleting individual GINS components variably affected the expression of the other GINS proteins. Notably, GINS4 depletion led to the most pronounced reduction in the levels of other GINS proteins (Supplementary Fig. 7e), whereas trace amounts of GINS4 remained detectable even upon depletion of GINS1, GINS2, or GINS3 (Supplementary Fig. 7e). These findings suggest that GINS4 may function as a central stabilizing factor within the GINS complex, likely contributing to its integrity and replication initiation activity. However, whether the role of GINS4 in A/B compartment regulation is mediated through the GINS complex as a whole remains to be determined and will require further investigation (see also Discussion).

To further assess whether GINS4 regulates compartment structure through the CMG helicase complex, we generated cell lines in which CDC45, another essential component of the CMG complex, can be conditionally depleted (Supplementary Fig. 7f). Similar to GINS4-KD cells, CDC45-KD cells exhibited impaired replication progression (Supplementary Fig. 7g), confirming effective disruption of replication initiation. However, despite clear G1-to-S arrest, the Hi-C profile of CDC45-KD cells remained largely indistinguishable from that of control cells (Supplementary Fig. 7h). These results indicate that replication arrest per se is not sufficient to induce A/B compartment defects and support the notion that GINS4 plays a specific and nonredundant role in the regulation of A/B compartment organization.

### Compartment defects are not artifacts of drug-induced cell cycle synchronization

One legitimate concern is that the extensive use of drugs for cell-cycle synchronization might have affected cell growth and, consequently, altered A/B compartment organization even after the drugs were removed. For example, as shown in Fig. 3e, treatment with nocodazole and 5-Ph-IAA delays the cell cycle. To determine whether the A/B compartment defects in GINS4-KD cells were due to drug-induced cellular stress and sickness, we treated asynchronous *Gins4*-mAID cells with 5-Ph-IAA alone and conducted time-course Hi-C on G1-accumulated, sorted cells (Supplementary Fig. 8a). The saddle plot profiles and compartment strengths of the 9- and 15 h 5-Ph-IAA-treated, synchronization-free G1 cells were similar to those observed in synchronized cells at the corresponding time points (Fig. 3e). PCA of saddle plot profiles also revealed that GINS4-depleted cells without nocodazole treatment gradually shaped an abnormal trajectory between 9 and 15 h (Supplementary Fig. 8b). These results clearly indicate that the loss of B-B interactions was caused by the depletion of GINS4 and not the combined use of cell-cycle synchronization drugs.

### B-compartment aggregates accumulate in the nucleus upon GINS4 depletion

**Fig. 4:**
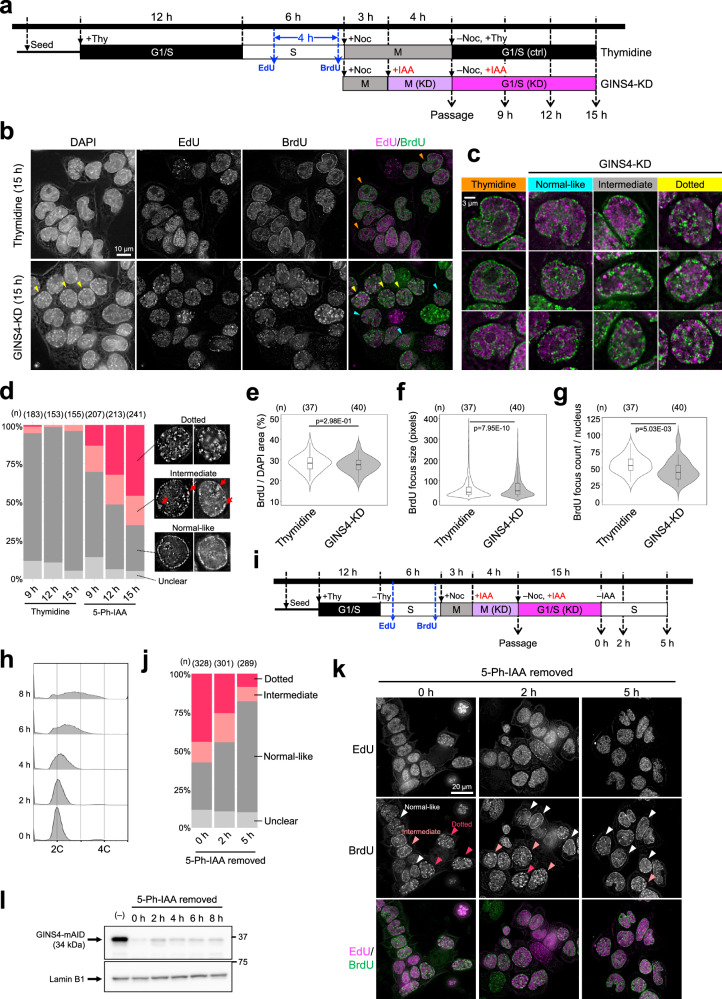
GINS4-depleted cells show nuclear aggregates of B-compartments. **a** Scheme of EdU/BrdU pulse-labeling in S-phase to visualize A/B compartments in the subsequent G1-to-S phase transition with or without GINS4 depletion. Noc, nocodazole; Thy, thymidine; IAA, 5-Ph-IAA. **b** Representative images of EdU/BrdU-stained cells with or without GINS4 depletion for 15 h. Representative data from at least three independent experiments with similar results are shown. Images within this panel are shown at the same magnification. Scale bar, 10 µm. Orange arrowheads, nuclei with typical BrdU signal patterns in the thymidine control; light-blue arrowheads, normal-like BrdU signal patterns in GINS4-KD; yellow arrowheads, dotted BrdU signal patterns in GINS4-KD. **c** Magnified images of representative BrdU signal patterns including nuclei corresponding to those indicated by arrowheads in (**b**). Images within this panel are shown at the same magnification. Scale bar, 3 µm. “Normal-like” nuclei show small dots at nuclear/nucleolar periphery. “Dotted” nuclei show large dots at nuclear/nucleolar periphery. “Intermediate” nuclei show both characteristics. **d** Gradual changes in BrdU nuclear foci patterns after 5-Ph-IAA mediated GINS4 depletion. BrdU spatial patterns were classified into four: dotted, intermediate, normal-like, as described in (**c**), and unclear ( = suboptimal signal quality). Thymidine treatment served as a control. Experiments were independently repeated at least three times with similar results; quantification shows nuclei counted from one representative experiment. Representative images are shown on the right. Red arrows indicate aggregated dotted signals in intermediate nuclei. **e**–**g** Quantification of BrdU signals in thymidine control and GINS4-KD cells from one representative experiment; experiments were independently repeated at least three times with similar results. *n* values indicate the number of nuclei analyzed. *P* values are indicated in the panels and were calculated using two-sided Wilcoxon rank-sum tests. Violin plots show the distribution of individual nuclei. For embedded box plots, center lines indicate medians, box bounds indicate first and third quartiles, and whiskers extend to the most extreme values within 1.5× the interquartile range. **e** BrdU signal area normalized to DAPI area. **f** BrdU focus size measured by pixel number. **g** BrdU foci count per nucleus. **h** Time-course cell-cycle profiles after 5-Ph-IAA removal by flow cytometry. Drug treatments were performed as described in Supplementary Fig. 11a. Cells were stained with Propidium iodide. **i** Experimental scheme to visualize B-compartment recovery in cells that entered S-phase upon 5-Ph-IAA removal after GINS4-KD-mediated G1/S arrest. **j** Gradual changes in the BrdU nuclear foci patterns after 5-Ph-IAA removal. Nuclei were classified in the same way as in (**d**). **k** Representative EdU/BrdU-staining patterns of nuclei after 5-Ph-IAA removal. Arrowheads in white, light-pink, and pink indicate normal-like, intermediate, and dotted nuclei, respectively. Images within this panel are shown at the same magnification. Scale bar, 20 µm. **l** Immunoblotting of nuclear GINS4 protein after 5-Ph-IAA treatment for 15 h followed by its removal (2–8 h). Lamin B1 was detected on a separate blot processed in parallel and used as a sample processing control. The experiment was independently repeated three times with similar results. Source data for (**d**–**g**, **j**) are provided as a Source data file.

Having successfully generated a cell line capable of manipulating compartments, we sought to further investigate the A/B compartment dynamics and their relationship to DNA replication. First, to directly observe the GINS4-KD-mediated A/B compartment defects inside the nucleus, we labeled early-replicating (A-compartment) and late-replicating (B-compartment) domains with different nucleotide analogs in S-phase and visualized their subnuclear positioning in the subsequent cell cycle after GINS4 depletion (Fig. 4a). Specifically, after G1/S arrest by thymidine, cells were released into S-phase, and we pulse-labeled early- and late-replicating DNA with EdU and BrdU, respectively. The labeled cells were again arrested at metaphase by nocodazole and then released while being depleted of GINS4 by 5-Ph-IAA. Finally, the effect of GINS4 depletion on subnuclear positioning of EdU and BrdU signals in the subsequent G1 was assessed (Fig. 4a and Supplementary Fig. 9a). To eliminate cells that failed to respond to the initial thymidine block, we analyzed only the EdU/BrdU double-stained nuclei.

In the thymidine-treated control cells, early and late replication foci were repositioned to the nuclear interior and nuclear/nucleolar periphery, respectively, as expected (Fig. 4b, c). In GINS4-KD cells, however, the nuclear organization of the BrdU-labeled late-S foci was markedly abnormal in most cells, which was also reflected in the DAPI signal pattern (Fig. 4b, c). Similar to the control cells, EdU-labeled early-S foci were observed uniformly in the nuclear interior without a specific pattern in GINS4-KD cells, meaning that we could not detect defects, if any, in A-compartment organization at this global scale. As there were various late-S foci patterns, we classified them into three categories (Fig. 4c, dotted, intermediate, and normal-like) at 9, 12, and 15 h after 5-Ph-IAA treatment and quantified the nuclear organization defects. This analysis revealed a gradual increase in the fraction of the dotted foci pattern in the absence of GINS4 (Fig. 4d), consistent with a dysregulation of B-compartment integrity observed by time-course Hi-C in G1/S-phase.

Unlike GINS4, depleting GINS1, GINS2, and GINS3 did not cause cell-cycle arrest (Supplementary Fig. 7d). However, it is possible that their depletion may still affect the 3D genome organization. To test this, we performed an experiment with a scheme identical to Fig. 4a except that the depleted factors were GINS1, GINS2, and GINS3. While we could reproduce the dotted abnormal pattern of late-S BrdU foci by GINS4-KD, depletion of GINS1, GINS2, or GINS3 did not produce such abnormal BrdU foci patterns (Supplementary Fig. 9b). These results suggest that GINS1, GINS2, or GINS3 by themselves individually are not essential for B-compartment integrity.

To rule out the possibility that these chromosome structural changes were induced by the extensive use of cell-cycle synchronization drugs, we performed a pulse-chase-pulse labeling experiment using EdU followed by BrdU on unsynchronized cells (Supplementary Fig. 9c). Although the number of EdU/BrdU double-labeled cells was low, which was expected without synchronization, we observed the characteristic dotted abnormal pattern of late-S BrdU foci in unsynchronized GINS4-KD cells. This confirms that the structural defects are not the result of drug-induced toxicity or cell sickness.

To better understand the B-compartment defects in GINS4-KD cells, we further characterized the BrdU late-S foci properties. As cells were on different focal planes, which made quantification difficult, we cropped each single-cell image focusing on its center focal plane and performed various BrdU signal measurements using the CellProfiler software^43^ (Fig. 4e–g). While the BrdU-occupied area (=late-S foci area) was comparable between control and GINS4-KD cells (Fig. 4e), the BrdU foci became significantly larger, and the focus number decreased in GINS4-KD cells (Fig. 4f, g). This suggests that individual late-replicating foci did not become decompacted, but rather they changed their relative subnuclear positioning and formed nuclear aggregates of B-compartment domains in the absence of GINS4.

A single stretch of a B-compartment domain contains multiple topologically associating domains (TADs), which are likely equivalent to individual replication foci^44^. Thus, we speculate that these nuclear aggregates of B-compartment domains are equivalent to B-compartment domains approaching the nuclear interior without changing their constituent TAD structure. Indeed, the number and the size of TADs identified was comparable between GINS4-KD and thymidine-treated cells (Supplementary Fig. 10a), especially within B-compartment regions (Supplementary Fig. 10b). Moreover, the insulation score profiles used to define TAD boundaries were also highly similar between the two conditions (Supplementary Fig. 10c; *R* = 0.91), indicating that overall, TAD organization is preserved in GINS4-KD cells. Such a view is consistent with the increased short-range A-B interactions (<10 Mb), decreased long-range B-B interactions (>10 Mb), and minimal changes in short-range B-B interactions (Fig. 3d, g). Increased short-range A-B interactions must reflect contacts between neighboring A and B compartment domains based on the Hi-C heatmap of GINS4-KD cells in G1 fraction (Supplementary Fig. 5c). Moreover, it is also consistent with the presence of large gaps between late-replicating foci at the nuclear periphery (Fig. 4c, Dotted nuclei).

### GINS4 recovery restores the B-compartment organization and, in turn, efficient DNA replication

We wondered whether the GINS4-KD-induced defects in B-compartment nuclear organization could be reversed when the GINS4 expression level is restored. To test this, we first removed 5-Ph-IAA from GINS4-depleted cells to allow GINS4 to accumulate and then observed whether the aggregated B-compartment foci were repositioned to the nuclear periphery and/or could initiate DNA replication (Supplementary Fig. 11a). After 5-Ph-IAA removal, cells started to initiate DNA replication, as assayed by flow cytometry measuring DNA content (Fig. 4h), and the nuclear organization of the late-replicating BrdU foci was restored at 2 and 5 h after 5-Ph-IAA removal (Fig. 4i–k).

Importantly, the cell number did not change significantly during GINS4 recovery (Supplementary Fig. 11b), which strongly suggests that nuclei with abnormal B-compartment foci organization at 0 h showed restoration of their nuclear organization. Notably, nuclear GINS4 expression level following 5-Ph-IAA removal did not fully recover to the level observed in untreated cells (nor did the expression levels of other GINS components), suggesting that even minimal levels of GINS4 expression are sufficient for nuclear reorganization and replication initiation (Fig. 4l and Supplementary Fig. 11c). Altogether, the compromised nuclear organization of B-compartment domains in GINS4-depleted nuclei is reversible and can be rescued even by partial restoration of GINS4.

**Fig. 5:**
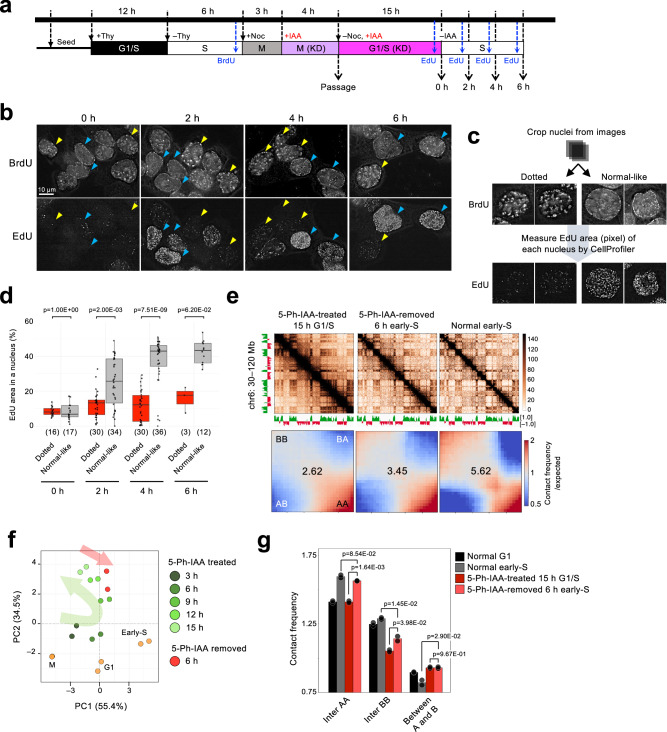
GINS4 recovery caused partial B-compartment restoration, which led to efficient DNA synthesis. **a** Scheme of late-S BrdU labeling in S-phase to visualize the B compartment after release from GINS4-KD-mediated G1/S-arrest. Cells were EdU-labeled for 30 minutes just before fixation to monitor DNA synthesis. Noc, nocodazole; Thy, thymidine; IAA, 5-Ph-IAA. **b** Representative BrdU/EdU nuclear foci patterns after 5-Ph-IAA removal. Yellow and blue arrowheads indicate nuclei with representative dotted and normal-like BrdU foci, respectively. The ratio of each foci pattern is shown in Supplementary Fig. 10d. **c** Scheme of EdU incorporation measurement of dotted and normal-like nuclei. **d** Changes in EdU-occupied area after 5-Ph-IAA removal in dotted and normal-like nuclei. Each dot indicates a single nucleus. *P* values are indicated in the panel and were calculated using two-sided Wilcoxon rank-sum tests between “Dotted” and “Normal-like” nuclei at each time point. Box plots show the median, first and third quartiles, and whiskers extending to the most extreme values within 1.5× the interquartile range. (**e**) Hi-C contact heatmaps and saddle plots for three populations: (left) 5-Ph-IAA-treated 15 h G1/S cells (identical to ‘GINS4-KD 15 h’ in Fig. 3E and Supplementary Fig. 5c), (center) 5-Ph-IAA-removed cells that moved to early S (“5-Ph-IAA-removed 6 h early-S”), (right) normal early-S cells sorted from asynchronous cells. Numbers at the center of the heatmaps indicate compartment strength. **f** A PCA plot generated from saddle plot data derived from Hi-C data of cells released from 5-Ph-IAA (indicated as red dots) and time-course Hi-C data obtained in Fig. 3e. See also Supplementary Fig. 10f for the actual FACS data. **g** Between-compartment contact frequency calculated by Pentad. Normal G1 and early-S represent G1 and early-S populations sorted from asynchronous cells, respectively. “5-Ph-IAA-treated 15 h G1/S” represents GINS4-KD-mediated G1/S-arrested (15 h) and sorted cells. “5-Ph-IAA-removed 6 h early-S” represents early-S cells sorted after 6-h release from GINS4-KD-mediated G1/S-arrest. See also Fig. 3e and Supplementary Fig. 10f for the actual FACS data. Bars indicate mean values and dots indicate individual biological replicates. *P* values were calculated using two-sided Student’s *t*-tests between “Normal G1” and “5-Ph-IAA-removed 6 h early-S”, and “5-Ph-IAA-treated 15 h G1/S” and “5-Ph-IAA-removed 6 h early-S”. Source data for (**d**, **g**) are provided as a Source data file.

The reversible nature of B-compartment organization before DNA replication motivated us to further dissect its relationship to the initiation of DNA synthesis. To do so, we optimized a protocol to observe B-compartment organization and DNA synthesis after GINS4 recovery in the same nucleus. We achieved this by labeling the late-S replication foci in the preceding S-phase with BrdU and then labeling the newly-replicated DNA in the following S-phase with EdU at different time points after GINS4 recovery (Fig. 5a). Although this was a single-labeling experiment of late-replicating DNA by BrdU, the ratio of nuclei with dotted BrdU signal patterns and its changes over time (Supplementary Fig. 11d) were similar to the EdU/BrdU double-labeling experiment in Fig. 4i, j.

Interestingly, by visual inspection, nuclei with reorganized B-compartment (i.e., late-S BrdU) foci could efficiently incorporate EdU, while the dotted nuclei could not (Fig. 5b). To quantify this, we focused on the dotted and normal-like BrdU signal nuclei from each time point and compared the EdU foci area of these nuclei using the CellProfiler software (Fig. 5c and Supplementary Fig. 11e). While the EdU foci area steadily increased over time in normal-like nuclei, this was not the case with the dotted nuclei (Fig. 5d), suggesting that restoration of B-compartment organization is a prerequisite for efficient DNA synthesis.

### Attenuated A/B compartmentalization is accompanied by defective RT regulation

B-compartment nuclear organization and DNA synthesis appeared to be restored by 5–6 h after 5-Ph-IAA removal (Fig. 4h, j, k). Consistent with this, Hi-C data of purified early S-phase population 6 h after 5-Ph-IAA removal showed re-establishment of B-B interactions (Fig. 5e and Supplementary Fig. 11f, 5-Ph-IAA-removed 6 h early-S). However, their A/B compartments were not as fully segregated and strengthened as in normal early S-phase cells (Fig. 5e and Supplementary Fig. 11f, Normal early-S). Compared to the GINS4-KD G1/S cells (Fig. 5e, 5-Ph-IAA-treated 15 h G1/S), the 6 h time point after 5-Ph-IAA removal (Fig. 5e, 5-Ph-IAA-removed 6 h early-S) showed only partial recovery of the compartment profile as assessed by saddle plot analysis, and the PCA plot indicated that the profile remained distant from the early S-phase state (Fig. 5f). In particular, the inter B-B and A-B interactions in “5-Ph-IAA-removed 6 h early-S” cells did not reach the contact frequency of normal early S-phase (Fig. 5g, gray and light red bars) and A-B interactions were still similar to the “5-Ph-IAA-treated 15 h G1/S” cells (Fig. 5g, dark red and light red bars). These results suggest that DNA synthesis can occur in nuclei with premature (i.e., incompletely segregated) compartment organization, which led us to further explore the possible impact of attenuated compartments on DNA replication, in particular, RT regulation.

**Fig. 6:**
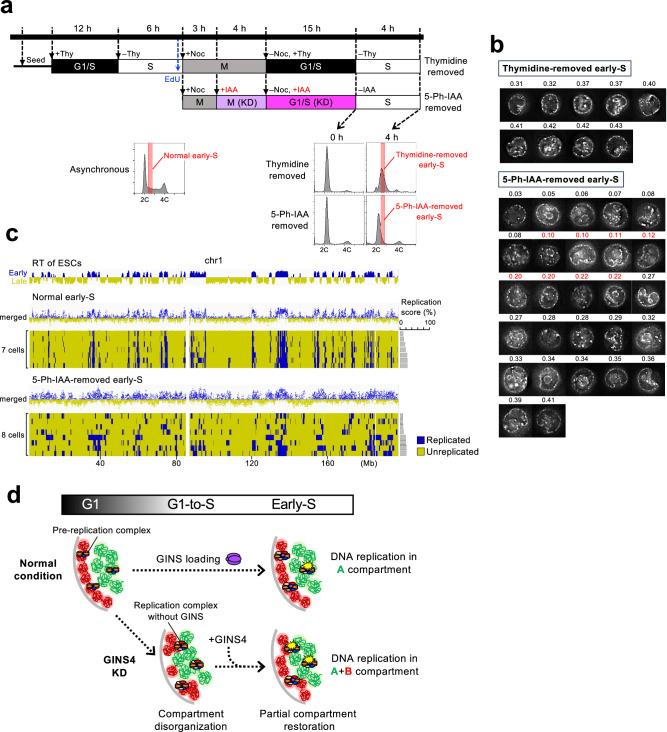
DNA synthesis with attenuated A/B compartments results in defective RT regulation. **a** Scheme of sorting B-compartment-labeled (late-S EdU) early-S population after release from thymidine or 5-Ph-IAA for EdU foci and scRepli-seq analyses. Lower panels show FACS plots showing the collection of early S-phase population from cells at 4 h after release from thymidine (“thymidine-removed early-**S**”) or 5-Ph-IAA (“5-Ph-IAA-removed early-S”). The 0 h plots are shown as references. In addition, we performed single-cell sorting of early-S cells from an asynchronous population (“normal early-S” FACS plot). Red highlighted populations were sorted. Noc, nocodazole; Thy, thymidine; IAA, 5-Ph-IAA. **b** Analysis of EdU (late-S) foci in individual cells from the “thymidine-removed early-S” and “5-Ph-IAA-removed early-S” samples shown in (**a**) prior to scRepli-seq in (**c**). Numbers on each panel indicate the replication score (%) of each cell, calculated by scRepli-seq analysis. Cells labeled with red numbers have a replication score of 10–25%. **c** Binarized scRepli-seq profiles of “normal early-S” cells from (**a**) or “5-Ph-IAA-removed early-S” cells observed under the microscope in (**b**), shown alongside with BrdU-IP RT profiles of mESCs (top) and merged log2-median scRepli-seq profiles (above each binarized profiles). Early-S phase cells were analyzed and ordered based on their percentage replication score. Each row of the binarized panels represents a single-cell profile (80-kb bins). Chromosome 1 is shown. Source data are provided as a Source data file. (**d**) A model of subnuclear compartment regulation during G1-to-S-phase transition and its impact on replication initiation, which is influenced by the presence or absence of GINS4 protein.

To evaluate the impact of compartment segregation “maturity” on RT, we monitored replication initiation genome-wide at 4 h after 5-Ph-IAA removal, when cells are undergoing A/B-compartment restoration (Supplementary Fig. 11d). Specifically, we combined single-cell DNA replication sequencing (scRepli-seq)^45^ with B-compartment visualization. We first labeled late-S replicating B-compartment DNA with EdU following the synchronization protocol described in Fig. 6a. After a 4 h release from thymidine (“thymidine removed”) or 5-Ph-IAA (“5-Ph-IAA removed”), we stained the cells with EdU and sorted the early S-phase cells by flow cytometry (Fig. 6a and Supplementary Fig. 12a; “thymidine-removed early-S” and “5-Ph-IAA-removed early-S”). The majority of cells after release from 5-Ph-IAA exhibited normal-like EdU pattern, characterized by small late-replicating foci decorating the nuclear periphery in a manner similar to cells released from thymidine (Fig. 6b). This observation supported the idea that replication occurred efficiently in cells whose B-compartment structure had been reorganized following 5-Ph-IAA removal (Fig. 5d).

Next, we manually picked the EdU-stained single cells under the microscope (Fig. 6b) and analyzed their RT by scRepli-seq (Supplementary Fig. 12a, “thymidine-removed early-S” and “5-Ph-IAA-removed early-S”). As a result, the “thymidine-removed early-S” control cells showed RT profiles similar to each other and their replication scores were greater than the “5-Ph-IAA-removed early-S” cells, which was consistent with their cell-cycle profiles (Fig. 6a, see FACS profiles; Supplementary Fig. 12b, see replication scores). Because of this, their direct comparison turned out to be difficult due to differences in their replication score range. Therefore, as a second control, we performed single-cell sorting of early S-phase cells from an asynchronous population and directly proceeded to scRepli-seq (Fig. 6a and Supplementary Fig. 12a, b, “normal early-S”). By scRepli-seq, the “normal early-S” cells showed gradual progression of DNA replication with increasing replication scores as previously reported^45^, and their replication scores were comparable to those of “5-Ph-IAA-removed early-S” cells (Fig. 6c and Supplementary Fig. 12b).

Unlike the “normal early-S” control cells, RT profiles of the “5-Ph-IAA-removed early-S” cells were not uniform, with some cells exhibiting replication initiation even in late-replication domains, especially in cells with replication scores below 25% (Supplementary Fig. 12b). Since cells with low replication scores may introduce noise into the RT analysis, we restricted the comparison to cells with replication scores ranging from 10–25% (Fig. 6c), which also ensures that GINS4 expression had been restored to a level sufficient to drive DNA replication in the “5-Ph-IAA-removed early-S” cells analyzed. This comparison clearly revealed a higher RT heterogeneity in the “5-Ph-IAA-removed early-S” cells than in the “normal early-S” controls. Although we cannot fully exclude the possibility that the observed RT heterogeneity reflects incomplete recovery of GINS4 (Fig. 4l; see also “Discussion”), it is tempting to speculate that attenuated A/B compartments may underlie this heterogeneity.

Although the “5-Ph-IAA-removed early-S” cells in general exhibited higher RT heterogeneity compared to the “normal early-S” cells, some cells showed relatively normal RT (Supplementary Fig. 12b). In particular, a larger proportion of cells with relatively normal RT was observed among those approaching mid-S (Supplementary Fig. 12b). In contrast, the earliest S-phase cells showed higher RT heterogeneity. Cells exhibiting substantial RT defects in early S-phase might have eventually ceased replication as S-phase proceeded. As a result, cells with only mild RT defects would become relatively enriched among those with higher replication scores. This selection bias could explain why cells with relatively normal RT were more frequently observed as cells approached mid-S. In summary, our unique experimental setup enabled us to explore the impact of attenuated A/B compartments on DNA replication, leading us to propose a model in which proper A/B compartment configuration is an important determinant of replication control (Fig. 6d).

## Discussion

While there have been a few reports on the analysis of RT and A/B compartment regulators^20,36,46^, to our knowledge, there has been no report of genome-wide screening for RT/compartment regulators, and our understanding of the molecular basis of A/B compartments has lagged behind. In this study, we developed a GFP-based RT reporter system and used it to perform a genome-wide CRISPR screen in mESCs, which identified genes required for early-S RT of the *Rex1* locus. Among the top hits were GINS4 and MCM6, components of the CMG helicase complex, which caused widespread RT defects upon knockdown. Consistent with a recent report^36^, A/B compartments were not affected by MCM6 knockdown. By contrast, GINS4 knockdown caused A/B compartment defects, namely loss of long-range B-compartment interactions and incomplete separation between A and B compartments. Our results indicate that GINS4 is essential for B-compartment integrity before replication initiation during the G1-to-S transition (Fig. 6d). Importantly, the GINS4-KD phenotype was not due to non-specific influences of the cell-cycle arrest drugs (Supplementary Fig. 8). Consistent with Hi-C data, GINS4-depleted cells exhibited nuclear aggregates that likely corresponded to multiple nearby B-compartment TADs along the chromosome, and these aggregates were dismantled by restoring GINS4 expression, indicating the reversible nature of nuclear compartments (Fig. 6d). Restoration of B compartments allowed the cells to restart DNA replication, suggesting that A/B compartment organization is a prerequisite for efficient DNA synthesis. However, replication in nuclei with incompletely restored A/B compartment organization resulted in RT defects, leading to replication initiation even in the normally late-replicating B-compartments during the early S-phase. Thus, we have revealed the importance of A/B compartmentalization in proper DNA replication and possibly RT control as well, suggesting an essential biological role for A/B compartments.

The 3D genome defects induced by GINS4-KD emerged during the G1-to-S transition, which is when the GINS complex is loaded onto the genome and is involved in replication initiation. This raises the question of whether GINS4 regulates 3D genome organization as a part of the GINS complex. When the other three subunits of the GINS complex, GINS1, GINS2, and GINS3, are depleted, the 3D genome defects are not observed. However, when GINS4 is depleted, GINS1, GINS2, and GINS3 are also substantially degraded, consistent with the idea that the stability of the GINS4 protein is closely linked to that of the entire GINS complex. Therefore, it is possible that 3D genome regulation represents a previously unrecognized function of the GINS complex, with GINS4 playing a central role. However, the compartment defects observed upon GINS4 depletion primarily affect B-compartment interactions, whereas replication initiation occurs predominantly within the A compartment immediately after S-phase entry. This spatial and temporal disconnect suggests that GINS4-mediated regulation of 3D genome organization is unlikely to arise from its canonical role within the CMG complex at the replication fork during ongoing DNA synthesis. The CDC45-KD experiment further supported this interpretation, as replication-impaired CDC45-KD cells did not show the dramatic A/B compartment defect observed in GINS4-KD cells. Collectively, these findings suggest that GINS4 regulates 3D genome organization through a mechanism distinct from its canonical role in DNA replication.

Although it may seem unexpected that replication-related factors have functions independent of replication control, there have been reports demonstrating the involvement of replication factors in various aspects of genome regulation beyond replication, including genome architecture, transcription, and genome stability^3,47^. Moreover, in multicellular organisms with large genomes and RT regulation, it remains surprisingly unclear exactly when and how the replication initiation complex is recruited onto the genome to ensure the proper progression of the RT program^48^. Our findings are particularly relevant to these unexplored aspects of replication regulation.

The analysis of RT in cells with insufficient A/B compartment segregation suggests that proper A/B compartmentalization plays an important role in RT regulation. However, incomplete restoration of GINS4 levels was observed after 5-Ph-IAA washout in GINS4-depleted cells (Fig. 4l), raising the possibility that the RT defects observed in Fig. 6 may result from insufficient GINS4 levels rather than impaired compartmentalization. Insufficient levels of replication factors can lead to genomic instability^49^. Another recent study reported that DNA replication is locally suppressed within TADs containing DNA double-strand breaks, suggesting that local inhibition of replication could influence RT regulation if DNA damage occurs during GINS4 depletion and/or recovery^50^. To address these possibilities, it will be necessary to perform a detailed dissection of the molecular functions of GINS4. While a separation-of-function GINS4 mutant (i.e., replication vs. 3D genome regulation) would be ideal for this purpose, we have yet to identify such mutants. Additionally, we note that some of the key Hi-C comparisons involved cell populations matched as closely as possible for cell-cycle stage but not identical in synchronization and recovery history. Although multiple orthogonal analyses supported the interpretation that compartment defects arise upon GINS4 depletion, we cannot formally exclude contributions from subtle differences in cell-cycle progression. Future studies using experimental systems that allow more precise control of cell-cycle transitions will help address this limitation. Nevertheless, our data demonstrate that disruption of A/B compartment organization at the G1/S transition severely compromises replication progression, indicating a tight functional coupling between 3D genome architecture and replication control. This intrinsic coupling likely explains why experimental separation of compartmentalization and replication functions of GINS4 has proven challenging.

Another major challenge is to elucidate the precise mechanism by which GINS4 regulates the 3D genome. Determining the genomic and nuclear localization of GINS4 will be essential, although our attempts thus far have been technically unsuccessful. One possibility is that GINS4 directly binds to the B compartment and enhances the B-B interaction strength. Alternatively, GINS4 may act indirectly without binding to the B compartment, perhaps functioning as a cell-cycle checkpoint component. However, because cells arrested at the G1-to-S transition by GINS4 knockdown are Geminin-positive (and thus have already entered S phase), it seems unlikely that this phenomenon is related to the G1/S checkpoint function.

In summary, based on the analyses of RT, Hi-C profiles, and late-replicating foci dynamics through manipulation of GINS4 expression, we propose that GINS4 plays an unexpected yet essential role in orchestrating A/B compartment organization during the G1-to-S transition. This organization appears to be essential for proper replication and contributes to RT control. While our data raise new questions to be addressed in the future, our findings represent an important breakthrough in our understanding of the A/B compartment regulation during the cell cycle, its relationship with RT regulation, and its biological significance.

## Methods

### Plasmid construction

Primer sequences and plasmid information are listed in Supplementary Data 1 and 2. The construction of gRNA/CAS9 expressing plasmid, pX459 (Addgene #48139), was performed as described previously^51^. The gRNA oligonucleotides are listed in Supplementary Data 3. For *Cas9* expressing cell line, pX459 was modified to express only gRNA by removing *Cas9* and T2A sequences, which is named pU6-sgRNAscaff-CBA-PuroR. The targeting vector to integrate the GFP cassette into the targeting regions was constructed as follows. First, pCAG-GFPd2 was obtained from Addgene (#14760), then the multicloning site, EcoRI-PstI-SalI-KpnI, was deleted for the later step. Next, the GFPd2 gene was modified to Emerald GFPd2 (EmGFPd2) with S72A-N149K-M153T-I167T mutations^25^. Then, the CAG-EmGFPd2 fragment was inserted into the HindIII-SalI region in pBluescript. We named it pBS-CAG-EmGFPd2. To integrate the insulator cassette into pBS-CAG-EmGFPd2, HSC1-HS4-D4Z4c-GiP^26^ was obtained from Addgene (#58541), then cloned HS4x2 fragment into the EcoRV site of pBS-CAG-EmGFPd2 using InFusion (TaKaRa, 639648). Next, PCR-generated HS4x2 fragment and D4Z4-c fragment were cloned into the 5’ site of the CAG promoter. We named this plasmid pBS-HS4-D4Z4c-CAG-GFPd2-HS4. After that, PCR-generated homology arms (~1 kb) were placed in 5’ and 3’ sites of the HS4-D4Z4c-CAG-GFPd2-HS4 fragment. The *Rosa26* targeting vector carrying the *Cas9* expression cassette was constructed by InFusion. PCR-generated *Rosa26* homology arms, EF1α promoter, and *Cas9* were inserted into pBluescript. For AID2 system, *Rosa26* targeting vector carrying the OsTIR1 (F74G) is kindly provided by Dr. Masato Kanemaki. AID-tag sequence was amplified from pMK344 or pMK290 (Addgene #121178 or # 72828, gifts from Dr. Masato Kanemaki) and cloned between 5’ and 3’ arms of the target site using InFusion.

### Cell culture

Male mouse ESC line used in this study is EGR-101, derived from C57BL/6 N^52^. Mouse ESCs were maintained at 37 °C with 5% CO_2_ on mouse embryonic fibroblasts (MEF) with KnockOut™ DMEM (ThermoFisher, 10829018), 1 mM Sodium Pyruvate (Nacalai, 06977-34), 1x MEM Non-Essential Amino Acids (Nacalai, 06344-56), 0.5 mg/mL penicillin-streptomycin (Nacalai, 09367-34), 2 mM L-Glutamine (Nacalai, 16948-04), 0.1 mM 2-mercaptoethanol (ThermoFisher, 21985023), Nucleosides solution (30 nM Adenosine, Guanosine, Cytidine, Uridine and 10 nM Thymidine), 20% FBS (BioWest, S1650-500) and 1000 U/mL mLIF (Nacalai, NU0012-2). For experiments, ESCs were expanded with NDiff 227 (Cellartis, Y40002), 1 μM PD0325901 (Wako, 162-25291) and 3 μM CHIR99021 (Wako, 034-23103), 0.1 mM 2-mercaptoethanol and 1000 U/ml mLIF on iMatrix-511 (Matrixome, 892012) coated dishes to remove MEF, and passaged every 2–3 days using trypsin/EDTA (Nacalai, 32777-44). For inducing degradation of an endogenous protein fused with mAID, 1 mM stock solution of 5-Ph-IAA in DMSO (a gift from Dr. Masato Kanemaki) was directly added to the culture medium at 1 µM concentration^33^.

### CRISPR-mediated genome editing

CRISPR guide RNAs (gRNAs) (listed in Supplementary Data 3) were designed using CRISPR direct (https://crispr.dbcls.jp/) and cloned into pX459. CRISPR-mediated genetic engineering was carried out as previously described^53,54^. A CRISPR plasmid with gRNA and a targeting vector were transiently transfected into mESCs cultured on MEF using Lipofectamine LTX&PLUS (ThermoFisher, 15338100). For ERCE deletion mutants, two CRISPR plasmids were transfected at the same time. Fifteen hours post-transfection, cells were selected with puromycin (1.0 µg/ml) for 48 h. After colonies appeared, they were picked and independently cultured in 96-well plates. Each ESC clone was split in duplicate; genomic DNA from one of the 96-well plates was extracted to screen the mutation by PCR. PCR fragments were also Sanger sequenced, and the selected clones were expanded for downstream experiments. For OsTIR1(F74G)-PGK-PuroR inserted cells, PGK-PuroR cassette was removed by Cre-loxP recombination. Cre-expressing plasmid (pCAGGS-Cre^55^) was transiently transfected, and recombinant mESC clones were used for further experiments. For the bulk-knockout (bulk-KO), we inserted a gRNA targeting each candidate gene identified from the CRISPR screening into pU6-sgRNAscaff-CBA-PuroR plasmid, which contains both a gRNA expression cassette and puromycin resistance gene cassette. After two days of puromycin selection, we labeled replicating DNA with BrdU for 2 h. We then collected the surviving cells, which were expected to express gRNA and harbor an indel (small insertions and deletion) at the targeted gene. The collected cells were immediately fixed in 75% ethanol and subject to BrdU immunoprecipitation (BrdU-IP). Note that bulk-KO cells are generated by a single gRNA transfection. Therefore, they are not clonal but instead consist of a mixture of various indels. As a consequence, the percentage of complete KO (functionally null mutant) cells in the population is unknown.

### Generation of genome-wide mutant libraries and screening

We performed CRISPR-gRNA genome-wide screening as described previously^29,56^. A total of 7.0–9.0 × 10^7^ cells expressing GFP and *Cas9* were transduced with a pre-determined volume of the genome-wide gRNA lentiviral supernatant that gave rise to 30% transduction efficiency measured by BFP expression. Three days after transduction, the cells were selected with puromycin for 3 days. Approximately 1 × 10^8^ ESCs were harvested 6 days post-transduction for FACS sorting. Cells were resuspended in culture medium with 20 × 10^6^ cells/ml concentration. DRAQ5 (Biostatus, DR50200) was added with 3 µl per 1 × 10^6^ cells concentration and incubated at 37 °C. After 10 min, cells were filtered through a cell strainer and sorted using a Sony MA900 cell sorter (purity mode). After sorting ~3 × 10^6^ cells per FACS gate in three independent experiments, genomic DNA was extracted and subjected to PCR using primers flanking the gRNA region. The PCR products were analyzed for gRNA abundance by next-generation sequencing (NGS).

### Illumina sequencing of gRNAs and statistical analysis

Illumina sequencing of gRNAs was conducted as described previously^29^. For each sample, 19-bp single-end sequencing was performed with the custom sequencing primer 5’-TCTTCCGATCTCTTGTGGAAAGGACGAAACACCG-3’. The number of reads for each guide was counted with an in-house script. Enrichment and depletion of guides and genes were analyzed using MAGeCK statistical package^31^.

### Sample preparation for RT profiling by BrdU-IP Repli-seq

We followed the BrdU-IP protocol as described^28,45^. ESCs were incubated in a medium containing 10 mM BrdU for 2 h before cell collection. After trypsinization, single-cell suspension was fixed in 75% ethanol. For FACS, we stained fixed cells with Propidium iodide (Nacalai, 29037) and used a Sony SH800 cell sorter (ultra-purity mode) to sort early- and late-S-phase cell population (at least 10,000 cells per fraction). We used a BioruptorII BR2012A (Sonic Bio) for genomic DNA sonication (high-output mode), with ON/OFF pulse times of 30 s/30 s for 5 times in ice-cold water. BrdU-incorporated DNA was immunoprecipitated using an anti-BrdU antibody (BD Biosciences Pharmingen, 555627). After BrdU-IP, immunoprecipitated DNA samples were subjected to whole-genome amplification using the SeqPlex kit (Sigma, SEQXE). NGS libraries were constructed from early- and late-replicating DNA after whole-genome amplification with an NGS LTP Library Preparation Kit (KAPA, KK8232) according to the manufacturer’s instructions and were subjected to NGS with the HiSeq X Ten system. The read numbers for each sample are provided in Supplementary Data 4.

### Sample preparation for Hi-C

For asynchronous cells, we followed the fixation method as previously described^57^. In brief, harvested cells were fixed with 1% formaldehyde in PBS for 10 min at room temperature, followed by quenching the reaction with 0.125 M glycine solution for 5 min. Fixed cells were washed with PBS and stored at –80 °C until the Hi-C experiment. For cell sorting, cells were fixed with the same method above and permeabilized with a small modification as described^58^. After permeabilization using 0.1% saponin with adjusted concentration to 1 × 10^6^ cells/mL PBS, 50 µg of Propidium iodide and 100 µg of RNase A for 1 × 10^6^ cells were added, and samples were incubated for 30 min at room temperature, protected from light. After washing once with cold 1×PBS, samples were resuspended in cold 0.5% BSA and 50 µg/mL Propidium iodide in PBS and immediately processed for FACS. FACS was performed using a Sony SH800 or Sony MA900 cell sorter (purity mode), and appropriate gates were set based on the relative levels of Propidium iodide. After FACS sorting, cell pellets were frozen in liquid nitrogen and stored at –80 °C.

### Hi-C and library preparation

Hi-C experiments were performed as previously described using 0.5–1 × 10^6^ fixed cells^57^. In brief, fixed nuclei were isolated and digested with DpnII (NEB, R0543), 5’ overhangs were filled in with a biotinylated nucleotide, blunt-ends were ligated, followed by reverse crosslinking overnight. The purified DNA (1–2 µg) was sonicated using Covaris (model: S220). The appropriate-sized sonicated DNA fragments were collected using AMPure XP beads (Beckman Coulter; first- and second-round size-selection for 150–400 bp fragments) and mixed with Dynabeads® M-280 Streptavidin (ThermoFisher, 11205D) and shaked for 15 min at 20 °C. The bead-bound DNA was washed and resuspended in 50 µl of buffer EB (QIAGEN, 19086). NGS libraries were prepared using the NGS LTP Library Preparation Kit according to the manufacturer’s instructions. Hi-C libraries were subject to paired-end sequencing (150 base pair read length) using HiSeq X Ten or NovaSeq X. The read numbers for each sample are provided in Supplementary Data 4.

### Computation associated with RT profiling of cell populations

We followed our established pipeline for BrdU-IP population RT analysis^45^. Mouse reference genomes, mm9 (chr1–19, chrX, chrM, and chrR (one copy of rDNA, GenBank BK000964.1)) assembly was used. After mapping, removing duplicate reads, and filtering the mm9 blacklist, we counted the reads of early- and late-S-phase BrdU-IP samples in sliding windows of 200- at 40-kb intervals and performed reads per million normalization. Then, the ratio of early-S-phase to total read counts ((early-S reads)/ (early-S reads + late-S reads)) was calculated for each bin and was further converted to make their distribution fit within ±1. This value was defined as the BrdU-IP RT score of each bin. We filtered out bins whose total read counts were within the bottom 5% of all bins. Hierarchical clustering was done using Ward’s method with Euclidean distance in R.

### Computations associated with Hi-C data processing

Hi-C data processing was done by using the 4dn-hic pipeline deposited in GitHub (https://github.com/4dn-dcic/docker-4dn-hic). The pipeline includes alignment (using the mouse genome, mm9) and filtering steps. After filtering valid Hi-C alignments, valid pairs were then binned into contact matrices at resolutions of 200 kb and then normalized using the cooler^59^. Output files (.cool files) were applied to generate Hi-C heatmaps and relative contact probability using the GENOVA tool^60^. The A/B compartment (Hi-C PC1) profiles (in 200-kb bins) in each chromosome were calculated, and saddle plots were generated using cooltools (https://github.com/open2c/cooltools). Intra- and inter-compartment strength and stratified compartments by genomic distance were calculated and visualized using the Pentad tool^38^. To analyze TAD numbers and sizes, we calculated insulation scores (window size = 10) and identified TAD boundaries using the GENOVA tool^60^. For this analysis, Hi-C contact matrices were converted into 40-kb resolution.cool files, which we used as input for insulation score computation and TAD calling.

### Protein detection

1–5 × 10^6^ mESCs were collected and lysed in RIPA buffer (25 mM Tris-HCl, pH 7.6, 150 mM NaCl, 1 mM EDTA, 1% NP40, 1% sodium deoxycholate, 0.1% SDS), followed by sonication using Bioruptor II BR2012A (high-output mode), with four cycles of “30 s ON/30 s OFF” pulses. After centrifugation, protein concentration was measured using Bradford assay with diluted samples. To isolate nuclear and cytoplasmic proteins, cell fractionation was performed with minor modifications to a previously described protocol^61^. Briefly, 4–7 × 10^6^ mESCs were lysed in CSK buffer A (10 mM HEPES, pH 7.9, 10 mM KCl, 1.5 mM MgCl_2_, 340 mM sucrose, 0.1% Triton X-100, 10% glycerol, 1 mM DTT, protease inhibitor cocktail (Sigma-Aldrich, P8340)) for 10 min on ice. The lysate was then centrifuged at 500 × *g* for 3 min at 4 °C, separating the supernatant fraction (containing cytoplasmic proteins) from the nuclei pellet. Nuclei were further lysed in CSK buffer B (3 mM EDTA, 0.2 mM EGTA, pH 8.0, 1 mM DTT, protease inhibitor cocktail) and incubated for 10 min on ice. The nuclear insoluble fraction was separated by centrifugation at 500 × *g* for 3 min at 4 °C. The resulting pellet was lysed in RIPA buffer and subjected to sonication using Bioruptor II BR2012A (high-output mode), with four cycles of “30 s ON/30 s OFF” pulses. After centrifugation, protein concentration was measured by Bradford assay with diluted samples.

For Western blotting, 10–20 µg of protein was mixed with 3x SDS sample buffer (190 mM Tris-HCl pH 6.8, 6% SDS, 30% glycerol, 6% 2-mercaptoethanol, 0.008% bromophenol blue) and heated at 95 °C for 5 min. Equal amounts of protein were loaded onto an Extra PAGE One Precast Gel (Nacalai) and transferred onto a 0.2 µm PVDF membrane (BIO-RAD, 1704272). The membrane was incubated with a primary antibody at 4 °C overnight, followed by incubation with an HRP-conjugated secondary antibody at room temperature for 1 h. Detection was performed using Amersham ECL Prime reagents (Merck), and images were acquired with a LumiographII EM (ATTO).

For Geminin staining, 1 × 10^6^ mESCs were fixed in 1% formaldehyde in PBS containing 10% FBS for 10 min, then quenched by 0.125 M glycine. After permeabilization in 0.1% Tween-20 in PBS containing 5% FBS (PBS-FT), cells were incubated with an anti-Geminin antibody (1:400, Abcam, ab175799) for 1 h on ice, washed several times with PBS-FT, and incubated with a secondary antibody for 30 min on ice. DNA was counterstained with 1 ng/µl FxCycle Violet solution for 30 min. Finally, cells were filtered through a cell strainer and analyzed using a Sony MA900 cell sorter.

### Antibodies

For protein detection, the following antibodies were used. Primary antibodies: anti-OsTIR1 (1:2000, a gift from Dr. Masato Kanemaki), anti-MCM6 (1:5000, SantaCruz, sc-393618), anti-ORC1 (1:1000, Cell Signaling, #4731), anti-GINS4 [SLD5] (1:500, GeneTex, GTX119779), anti-GINS1 [PSF1] (1:300, Abcam, ab181112), anti-GINS2 [PSF2] (1:1000, Proteintech, 16247-1-AP), anti-GINS3 (1:500, Proteintech, 15651-1-AP), anti-Geminin (1:400, Abcam, ab175799), anti-GAPDH (1:8000, Abcam, ab8245), anti-alpha TUBULIN (1:10000, Abcam, ab7291), anti-Vinculin (1:500000, Sigma, V9131). Secondary antibodies: anti-rabbit IgG HRP (ThermoFisher, 65-6120), anti-mouse IgG HRP (ThermoFisher, 62-6520). All secondary antibodies were used at a 1 in 5000 dilution with TBST containing 5% skim milk.

### mESC synchronization and GINS4 degradation

For the G1- and S-KD Hi-C, and time-course Hi-C experiments, Gins4-mAID mESCs were first synchronized in metaphase for 10 h with 50 ng/ml Nocodazole. 5-Ph-IAA was added at 6 h time point to degrade GINS4 in metaphase. Nocodazole was washed away with 2 times PBS wash, and then 1 µM 5-Ph-IAA or 2.5 mM thymidine was added to the culture medium for GINS4-KD or thymidine-treated control, respectively. For S-KD, cells were cultured without drug for 6 h to pass G1 phase, and 5-Ph-IAA was added to degrade GINS4 in S phase. For time-course Hi-C sampling, cells were fixed with 1% formaldehyde in PBS containing 10% FBS at each time point. For microscopic imaging of pulse-labeled DNA, mESCs were synchronized at G1/S boundary with thymidine for 12 h, and then released into S-phase by washing thymidine away after 2 times PBS washes. After 2 h, cells were treated with 20 µM EdU for 10 min at 37 °C to label the early-S replicating DNA, and then EdU was washed away with 2 times PBS washes. After another 4 h, cells were treated with 15 µg/mL BrdU for 10 min at 37˚C to label the late-S replicating DNA. After labeling, medium was replaced with nocodazole-containing medium to arrest cells at metaphase, followed by GINS4 degradation with 5-Ph-IAA treatment for 4 h. Metaphase cells were seeded to glass bottom dishes and released into subsequent G1 phase with thymidine- or 5-Ph-IAA-containing medium, then fixed with 4% paraformaldehyde in PBS at each time point. For 5-Ph-IAA release, cells were washed with PBS two times and cultured with fresh medium. To observe DNA synthesis after 5-Ph-IAA release, EdU was added 30 min before the fixation time points. Cell cycle progression was analyzed using FlowJo™ software (version 10.9.0; BD Life Sciences).

### EdU/BrdU staining

EdU/BrdU-labeled fixed cells on the glass bottom dish were washed with PBS. EdU staining was performed using Click-iT Plus EdU Alexa Fluor 594 Imaging Kit (Invitrogen). After EdU staining, samples were incubated with 2 M HCl for 30 min at room temperature and subsequently neutralized with 0.1 M sodium borate buffer (pH 9.0) for 15 min. After PBS washes, samples were treated with mouse anti-BrdU (1:100, Abcam, ab8039) primary antibody for 1 h at room temperature, washed several times in PBS, and incubated with Alexa Fluor 488 donkey anti-mouse IgG (H + L) (1:200, Invitrogen, A21202) secondary antibody for 1 h at room temperature. Nuclei were stained by DAPI and observed using DeltaVision Olympus IX71 equipped with Olympus PlanApo x60 1.42 numerical aperture (NA) oil objective, using the standard SoftWoRx acquisition software (v.6.5.2).

### Single-cell selection of EdU-labeled cells for scRepli-seq

Late-replicating DNA was pulse-labeled with EdU at the 6 h time point after thymidine release, following the same schedule as used for EdU/BrdU imaging. Immediately after the EdU pulse labeling, the medium was replaced with a nocodazole-containing medium to induce mitotic arrest. After 15 h of treatment with either 5-Ph-IAA or thymidine, the drugs were removed, and the cells were cultured with normal medium for 4 h. At each time point, cells were collected and fixed in 75% ethanol for FACS. Prior to sorting, we stained EdU using the Click-iT Plus EdU Alexa Fluor 594 Imaging Kit (Invitrogen) and stained DNA with FxCycle™ Violet Stain (Invitrogen). Cells in early S-phase were sorted using a Sony MA900 cell sorter (ultra-purity mode). Sorted cells were distributed into PBS drops on a glass-bottom dish at low density (~20 cells per drop), and their EdU signals were observed using a DeltaVision Olympus IX71 equipped with an Olympus PlanApo ×60 1.42 numerical aperture (NA) oil objective. After observation, we manually collected a single cell with a mouth pipette under the microscope and transferred it into 8-well PCR tubes containing 6 µl of cell lysis buffer (72 μl H_2_O, 0.5 μl of 10 mg/ml Proteinase K [Sigma, P4850], and 8 μl of 10× single-cell lysis and fragmentation buffer [Sigma, L1043]) for scRepli-seq, following a previously described protocol with modifications^62^. Sample preparations were based on Takahashi et al.^45^. In brief, cells in lysis buffer were incubated at 55 °C for 1 h and then at 99 °C for 4 min for gDNA isolation and fragmentation. For scRepli-seq analysis, we analyzed all cells unless there was an accidental damage to the cell/sample. The subsequent whole-genome amplification process (SeqPlex enhanced DNA amplification kit, Sigma-Aldrich, SEQXE) and next-generation sequencing (NGS) library construction (NGS LTP library preparation kit, KAPA, KK8232) were performed according to the manufacturer’s instructions. Finally, the samples were processed for NGS on the Illumina NovaSeq X.

### Computation associated with RT profiling of single cells

We followed our established pipeline for scRepli-seq analysis^45,63^. In brief, following NGS, raw scRepli-seq FASTQ files were processed for adapter trimming of both Illumina and SEQXE adapters and then mapped to the mm9 reference genome. Because mESCs tend to exhibit karyotype abnormalities on chr 8 and chr 11, we excluded these chromosomes from the reference genome and used the mm9 assembly consisting of chr1–7, 9, 10, 12–19, chrM, and chrR (one copy of rDNA, GenBank BK000964.1). Next, we filtered out the duplicated reads and reads that overlapped with the mm9 blacklist regions. For quality control of scRepli-seq data, we applied MAD-score-based screening to filter out cells with problematic data (i.e., cells with MAD scores of 0 or >1.0). More than 90% of cells in each sample passed these criteria. To generate log2[median] single-cell RT profiles, we counted the reads in sliding windows of 200 kb at 40 kb intervals after normalizing S-phase data with AneuFinder’s correctMappability command based on G1 control without karyotype defects. Binarization was performed using 80 kb windows with the findCNVs command in AneuFinder, as described previously^45^. For early S-phase cells, binarization was conducted in 1-somy mode (i.e., the default copy number is “unreplicated”). Finally, the percentage replication scores (i.e., the percentages of genomic bins that have completed their replication) were calculated from the binarized scRepli-seq data as described previously^45^.

### Statistics and reproducibility

Unless specified in the legends, the western blot and EdU/BrdU staining experiments were performed at least three times with similar results. All Hi-C and Repli-seq analysis were performed for two biological replicates.

### Reporting summary

Further information on research design is available in the Nature Portfolio Reporting Summary linked to this article.

## Supplementary information

Supplementary Information

Description of Additional Supplementary Files

Supplementary Data 1-4

Reporting Summary

Transparent Peer Review file

## Source data

Source Data

## Data Availability

All next-generation sequencing data in this study have been deposited in NCBI’s Gene Expression Omnibus and are accessible through GEO Series accession number GSE245938 [https://www.ncbi.nlm.nih.gov/geo/query/acc.cgi?acc=GSE245938]. All cell lines generated in this study are available from the corresponding authors upon request. Source data are provided with this paper.
