# Supplementary material for: Nuclear compartmentalization at the G1/S transition plays a key role in DNA replication control
Source: Nat Commun. 2026 Aug 10;17:6961. doi: 10.1038/s41467-026-75264-6 (PMC13458202; doi:10.1038/s41467-026-75264-6)
Supplement: Supplementary file 1 [file 41467_2026_75264_MOESM1_ESM.pdf]

Supplementary Note 1 | Rationale for using thymidine-treated cells as a control for GINS4-KD cells

Once we found that GINS4-depleted cells exhibited defects in 3D genome organization and cell-cycle arrest within the G1 phase (i.e., at 2C DNA content), it became clear that selecting an appropriate control was inherently challenging. The 2C DNA content alone does not define a precise cell-cycle stage, and G1 represents a dynamic period during which the 3D genome is progressively re-established following mitosis. We were fully aware of this complexity and therefore performed multiple complementary analyses to define the precise arrest point of GINS4-KD cells and to identify the most appropriate reference condition.

As shown in Figures 3E and 3F, principal component analysis (PCA) of time-course Hi-C data from GINS4-KD cells released from nocodazole-induced metaphase arrest revealed that these cells progressed through the G1 phase approximately 3–6 hours after mitotic exit, similar to thymidine-treated cells. During this interval, GINS4-KD cells established A/B compartments to a degree comparable to thymidine-treated cells (Fig. 3E,F), indicating that GINS4 is dispensable for the initial establishment of A/B compartments during early G1. However, beyond this stage, GINS4-KD cells failed to undergo further compartment maturation. These results indicate that GINS4-KD cells are not arrested in early G1 but rather at the G1/S boundary, corresponding to a late G1/early S-phase state. This conclusion was corroborated by the observation that GINS4 depletion in G1-arrested cells via INK-128 treatment did not alter their Hi-C profiles (Fig. 3H,I). Therefore, using a bulk population of G1-phase cells as a control would be inappropriate, as such a population includes early G1 cells, in which GINS4 depletion does not affect A/B compartment organization.

Based on these observations, we reasoned that the most appropriate control should consist of cells that retain GINS4 yet are arrested at the G1/S boundary without undergoing replication. Among the available options, thymidine-treated cells fulfill these criteria. Indeed, thymidine-treated cells arrest at 2C DNA content in a Geminin-positive state (Fig. S6A; Geminin serves as an S-phase marker). PCA of time-course Hi-C data from thymidine-treated cells (to which thymidine was added at the time of release from nocodazole arrest) demonstrated that these cells progressed through G1 within 3–6 hours after mitotic exit (Fig. 3E,F) and subsequently remained arrested in an early S-like configuration without detectable replication (Fig. 3F, Thymidine 9–15 h time points). Thus, the 9–15 h time points in the PCA trajectory represent the 3D genome configuration of thymidine-arrested cells at the G1/S boundary.

In contrast, GINS4-KD cells began to exhibit compartment abnormalities before reaching the early S-phase position on the PCA trajectory (Fig. 3E,F), indicating that 3D genome defects emerge at a cell-cycle stage slightly earlier than that represented by thymidine-arrested cells. While thymidine-treated cells may not perfectly mirror the precise arrest state of GINS4-KD cells, precise synchronization of cells at an identical late G1/early S-phase position remains technically challenging with currently available methodologies. On this basis, we consider thymidine-treated cells to provide the closest experimentally attainable approximation for interpreting the effects of GINS4 depletion on 3D genome organization.

Supplementary Figure 1

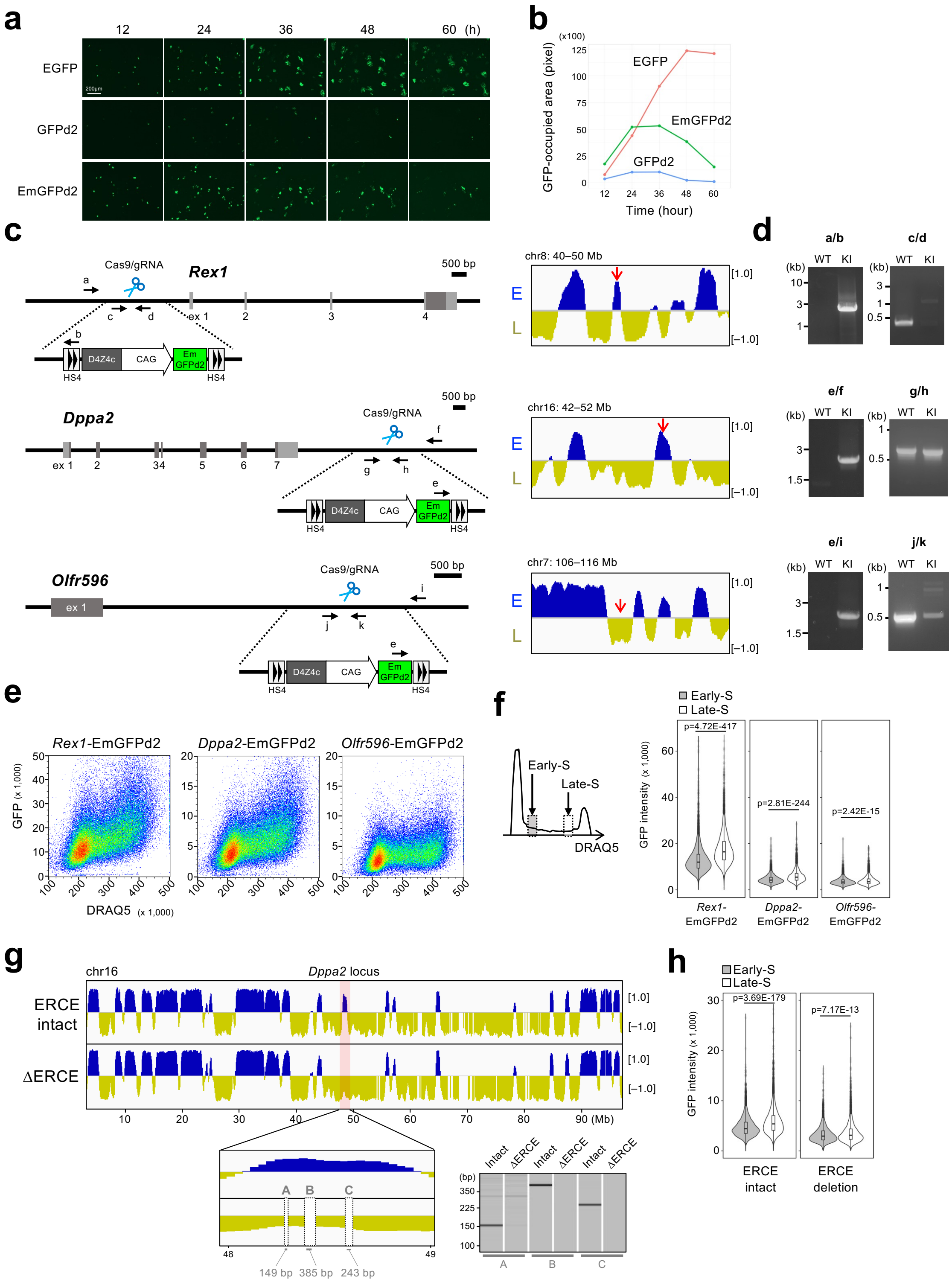

Supplementary Figure 1. Establishment of a GFP-based RT reporter system in mESCs
(a) Time-lapse imaging of mESCs after transient transfection of EGFP-, GFPd2- and EmGFPd2-expression vectors. Time interval is 12 hours. **(b)** Quantification of GFP-occupied area in each

image shown in (a). **(c)** Strategies for knock-in of EmGFPd2 expression cassette including HS4 and D4Z4c insulators into the intron region upstream or downstream of *Rex1*, *Dppa2* and *Olfr596* genes. Target sites for CRISPR/Cas9 are shown by scissors and primers. Black arrows represent PCR primers for genotyping. Early (E) and late (L) Repli-seq profiles of each locus is shown on the right. Red arrows indicate the EmGFPd2-inserted regions. **(d)** Knocked-in fragments were confirmed by genotyping PCR using primer sets targeting a region spanning from the inside of the EmGFPd2 cassette to the outside of homology arms (left: a/b, e/f, and e/i). Primer sets designed to include the CRISPR/Cas9 target sites were used to determine whether the GFP insertion was heterozygous or homozygous (right: c/d, g/h, and j/k). *Rex1*-EmGFPd2 cell line is a homozygous knock-in, *Dppa2*- and *Olfr596*-EmGFPd2 cell lines are heterozygous knock-ins. WT lane exhibits a control band amplified from wild-type C57BL/6 mouse genome. **(e)** FACS plots of *Rex1*-, *Dppa2*- and *Olfr596*-EmGFPd2 cell lines. Y-axis is adjusted to show similar cell populations between homozygous (*Rex1*-EmGFPd2) and heterozygous (*Dppa2*-EmGFPd2 & *Olfr596*-EmGFPd2) knock-ins. **(f)** Comparison of GFP intensity between early- and late-S-phase cell populations. Each cell population is gated based on the DRAQ5 intensity and the GFP intensity was quantified by FACS (left). On the right are violin plots of GFP intensity from gated cell populations in the three cell lines. Welch's t-test was performed to compare the early- and late-S populations. **(g)** RT profiles of ERCE-intact and ERCE-deleted cell lines on chromosome 16. Red highlighted area indicates the *Dppa2* locus. Three ERCEs (A, B, C) are described in the magnified RT profile below. Genotyping PCR was performed with three primer sets designed for each ERCE to ensure that all three ERCEs were deleted in the ΔERCE mutant. **(h)** Comparison of GFP intensity between the early- and late-S-phase cell populations in ERCE-intact and ERCE-deleted cell lines, gated in the same way as in (f). Welch's t-test was performed to compare the early- and late-S populations.

Supplementary Figure 2

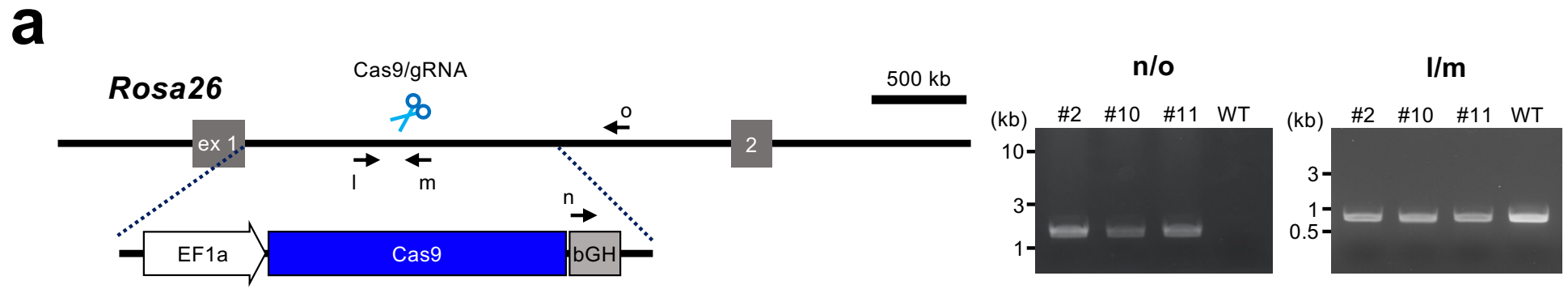

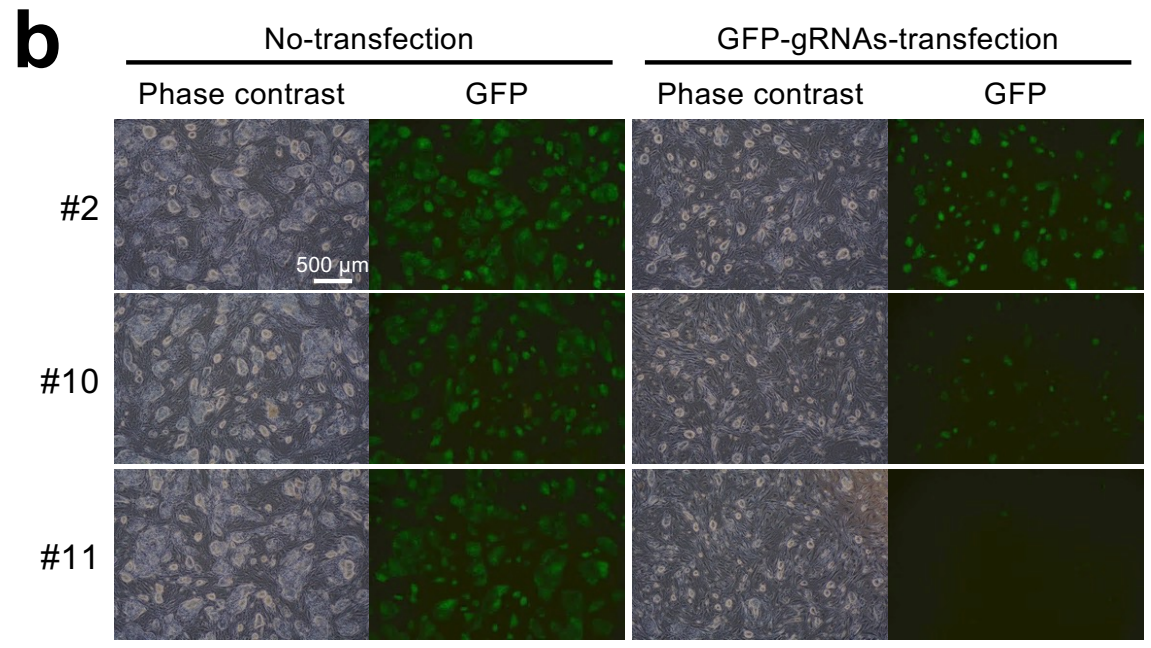

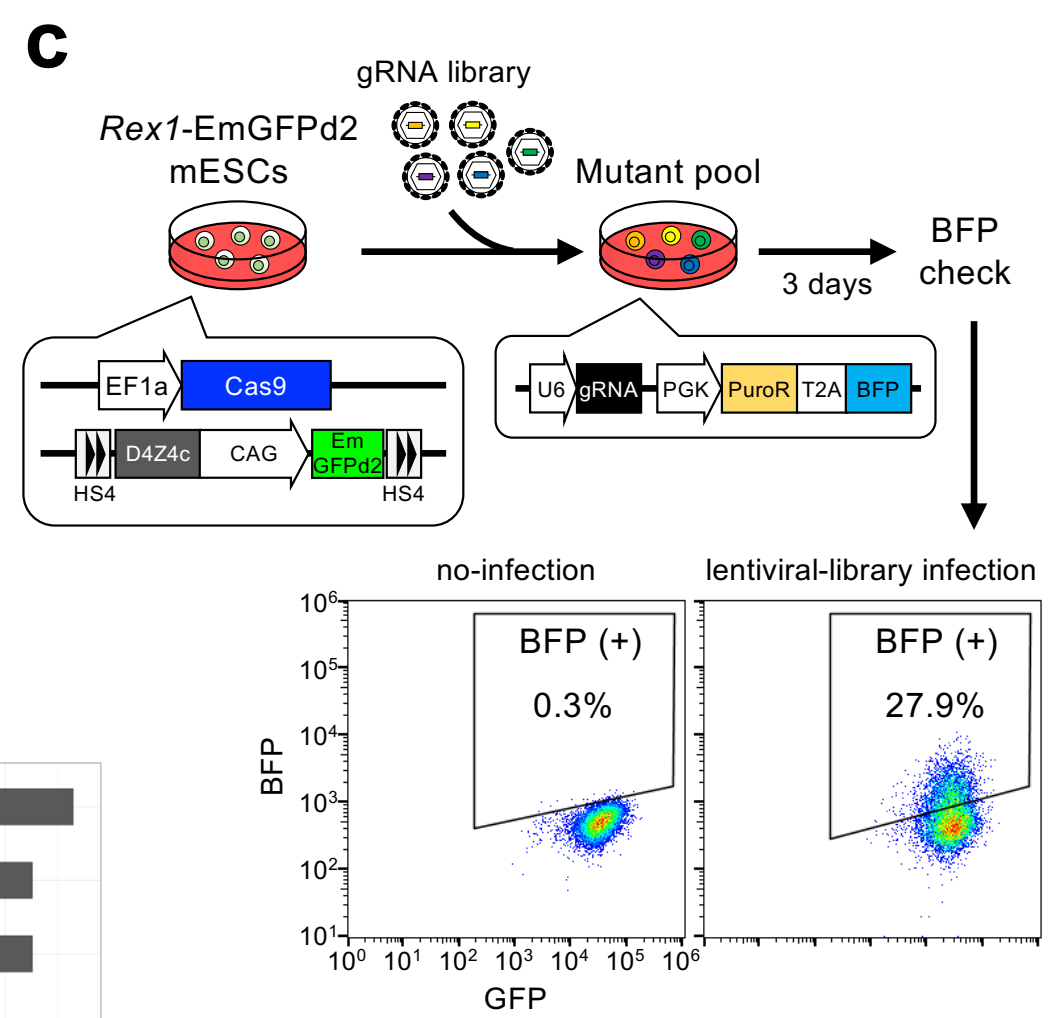

d

Rank	Gene	*P*-value
12	*Prim1*	2.63E-07
50	*Tbcc*	2.63E-07
57	*Tardbp*	2.63E-07
64	*Anapc11*	2.63E-07
87	*Pold2*	1.84E-06
92	*Gins1*	1.84E-06
95	*Ddb1*	2.37E-06
97	*Gins4*	2.37E-06
119	*Topbp1*	1.40E-05
135	*Hal*	3.19E-05
146	*Mcm6*	5.08E-05
152	*Aars2*	5.61E-05
163	*Atp6v0b*	7.40E-05
164	*Gins3*	7.61E-05
179	*Ddx39b*	1.10E-04
185	*Pfn1*	1.30E-04
187	*Orc1*	1.32E-04
190	*Haus1*	1.43E-04
191	*Rfc2*	1.44E-04

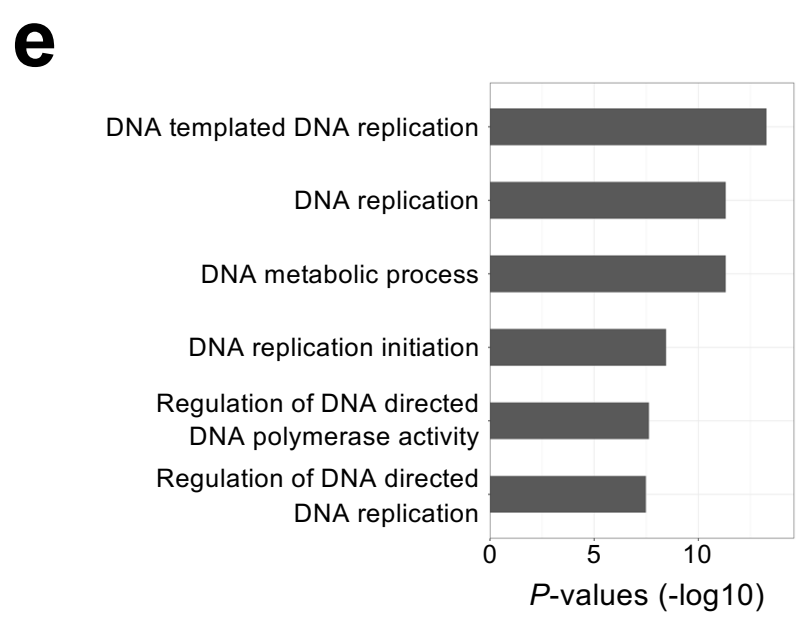

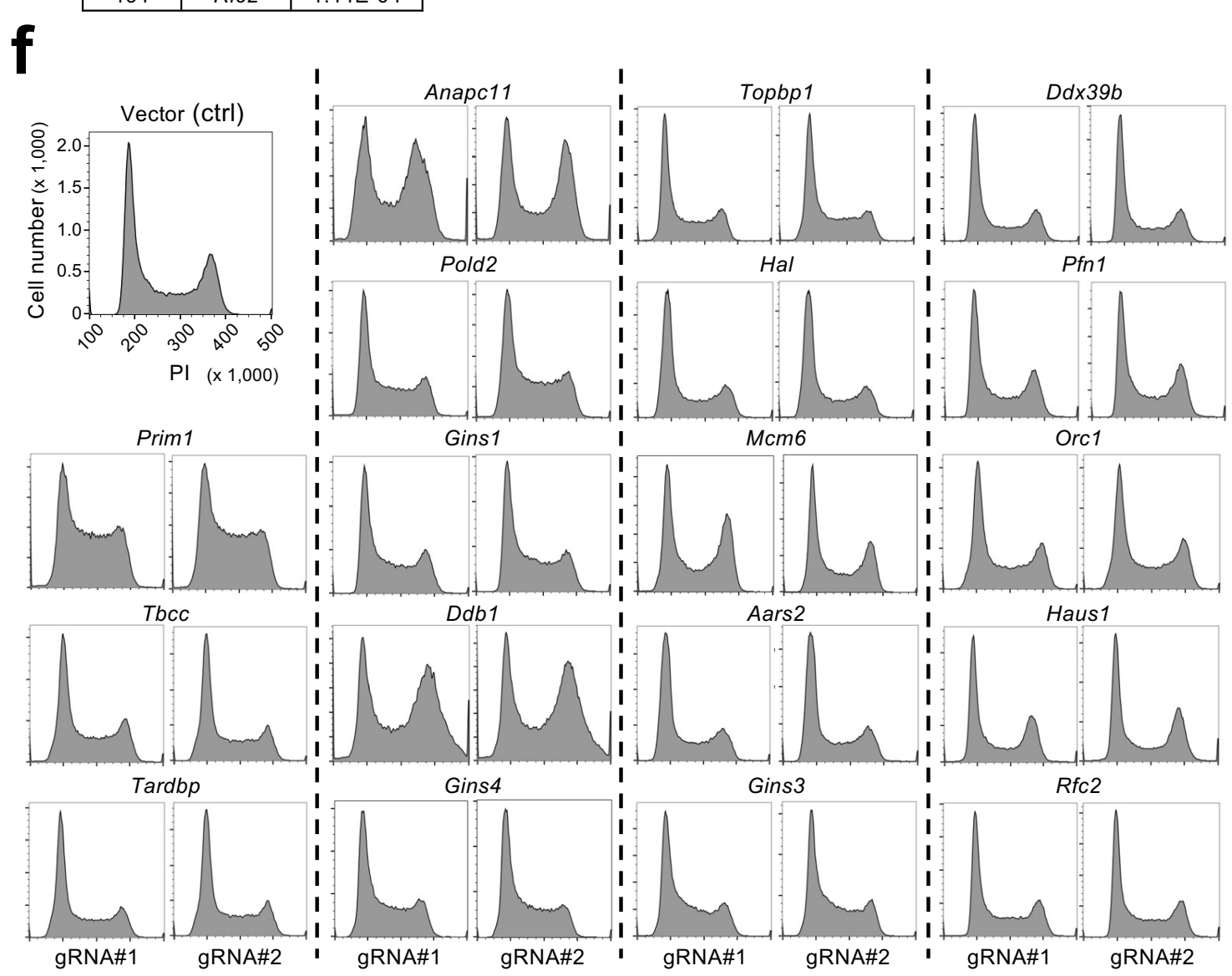

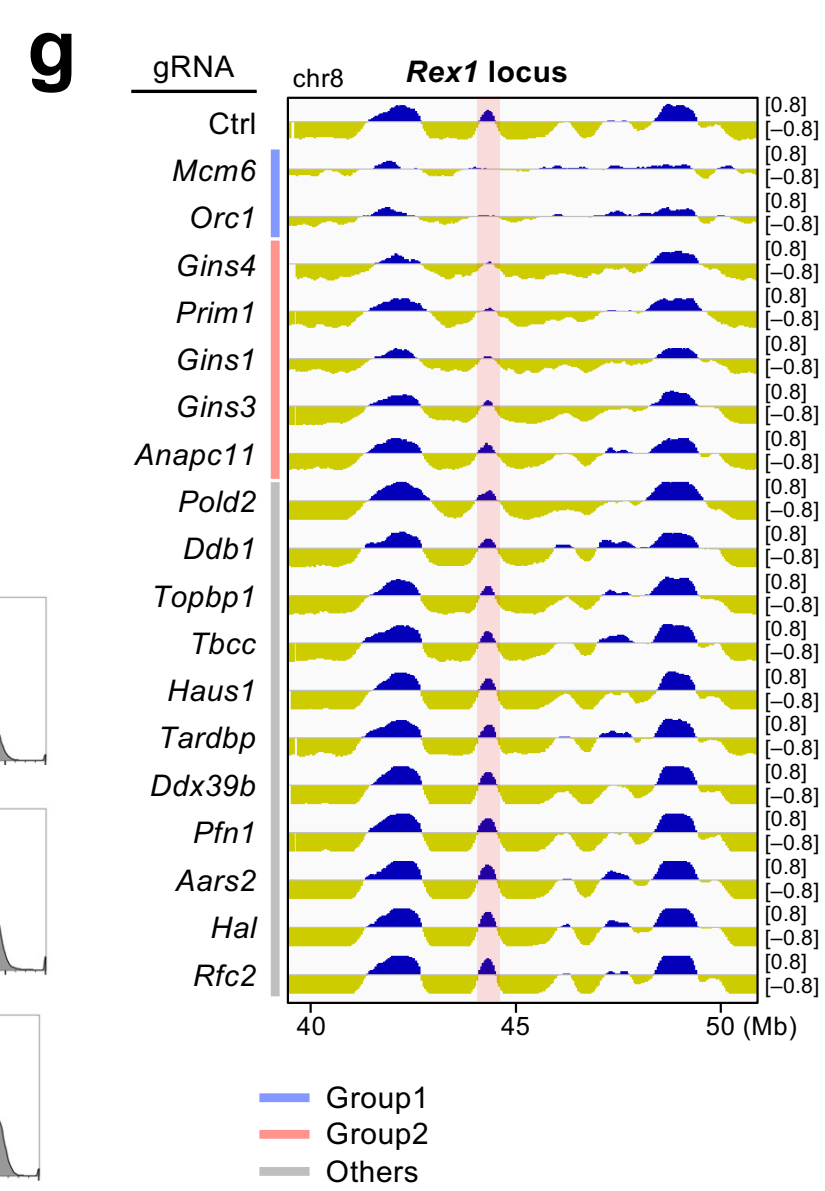

Supplementary Figure 2. RT regulators are successfully identified by CRISPR screening
(a) Knock-in strategy for Cas9 expression cassette into the *Rosa26* locus (left). Target sites for CRISPR/Cas9 are shown by scissors and primers. Black arrows represent PCR primers for genotyping. Primer set n/o is to confirm the insertion of the Cas9 fragment, and l/m is to check whether the knock-in is homozygous or heterozygous (right). **(b)** Cas9 activity checked by transfection of gRNAs targeting the GFP sequence. Decline of GFP fluorescence indicates efficient Cas9 activity. **(c)** Titer check of lentiviral library used for the CRISPR-screen. gRNA library with BFP expression cassette is induced into Cas9-expressing *Rex1*-EmGFPd2 mESCs, and the ratio of BFP positive cells are examined by FACS. **(d)** Top 19 candidates filtered from top 200 genes enriched in the GFP-low gate of Figure 1E. Genes are ranked by the MAGeCK (*31*) pipeline. **(e)** GO term enrichment analysis for biological processes of top 19 genes performed by GSEA. **(f)** Cell-cycle profiles of bulk-KO mutants detected by FACS based on DNA content (PI staining). Two gRNAs were individually transfected for one gene. As the transfection of *Atp6v0b*-targeting gRNA failed, we omitted *Atp6v0b* gene from further analysis. **(g)** RT profiles of bulk-KO mutants around the *Rex1* locus (chromosomes 8). Each row shows an averaged RT profile obtained from two bulk-KO mutants made by the two gRNAs.

Supplementary Figure 3

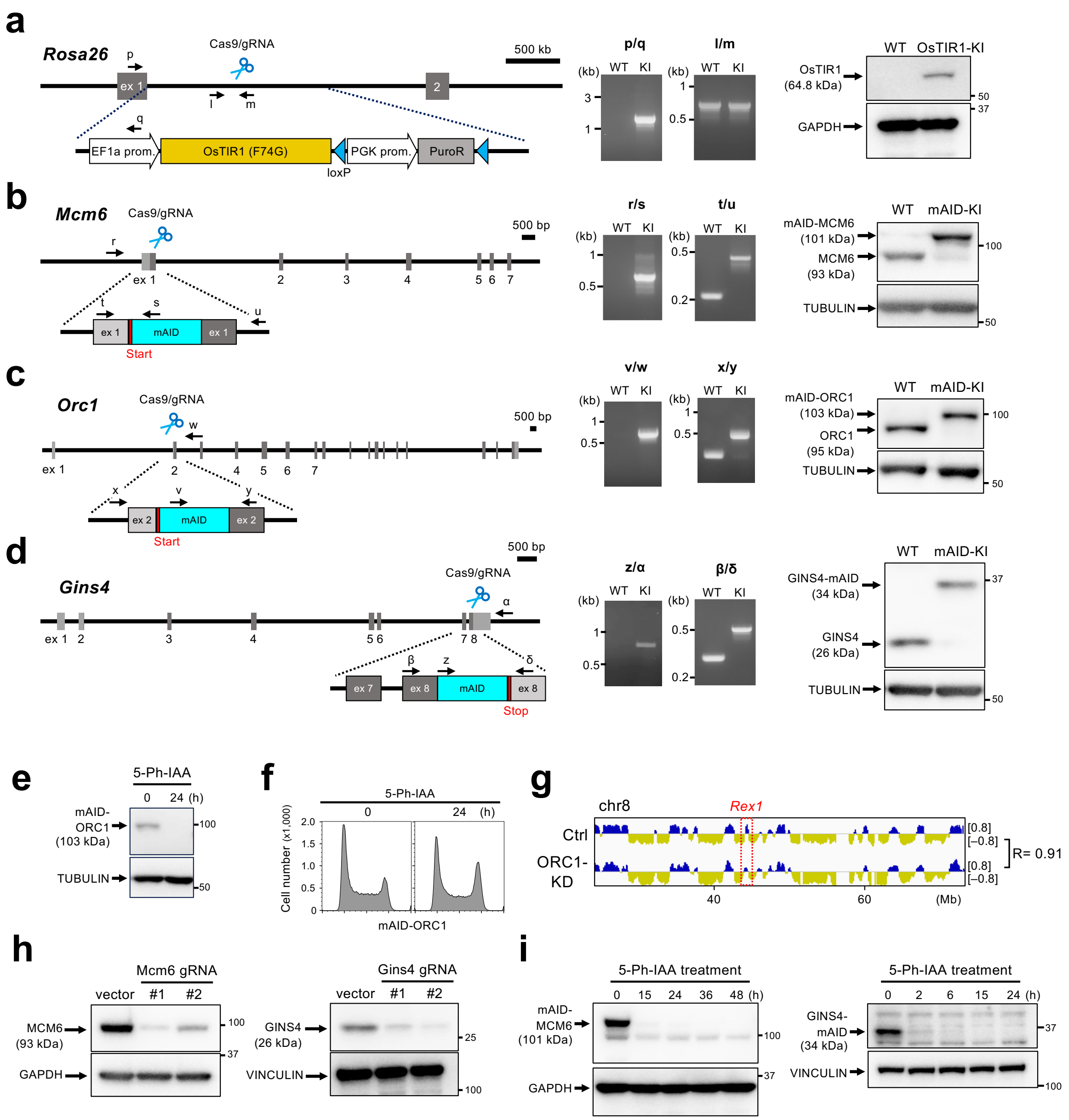

Supplementary Figure 3. Establishment of an AID2-based KD system

(a) (Left) Knock-in strategy for OsTIR1 (F74G) expression cassette into the *Rosa26* locus. Target sites for CRISPR/Cas9 are shown by the scissors. Primers for genotyping are shown by the black arrows and loxP sites are shown by the blue triangles. Primer set p/q is to confirm the insertion of OsTIR1 (F74G) fragment, and l/m is to check whether the knock-in is homozygous or heterozygous. (Center) Genotyping PCR. WT lane exhibits a control band amplified from wild-type C57BL/6 mouse genome. (Right) Expression of OsTIR1 (F74G) protein was confirmed by immunoblotting. GAPDH is used as a loading control. **(b–d)** Knock-in strategy for an AID tag into the N-terminus of *Mcm6, Orc1*, and C-terminus of *Gins4*. Biallelic insertion of AID tags were confirmed by genotyping PCR (center) and immunoblotting (right). TUBULIN is

used as a loading control. **(e)** ORC1 protein expression in the mAID-*Orc1* cell line following a 24-hour 5-Ph-IAA treatment. TUBULIN serves as the loading control. **(f)** Cell-cycle profiles of mAID-*Orc1* cells before and after 5-Ph-IAA treatment analyzed by flow cytometry after staining with propidium iodide. **(g)** RT profiles of mAID-*Orc1* cells with (ORC1-KD) or without (Ctrl) 5-Ph-IAA treatment. Chromosome 8 is shown. Red dotted box indicates the position of the early-replicating *Rex1* locus. Each row shows an averaged RT profile obtained from two replicates. Pearson's correlation coefficient values are shown on the right. **(h and i)** Comparison of protein expression levels between bulk-KO mutants and auxin-mediated KD of MCM6 and GINS4. Proteins were extracted after gRNA transfection (h, see Methods) or 5-Ph-IAA treatment (i) and were used for immunoblotting. GAPDH and VINCULIN were used as loading controls.

Supplementary Figure 4

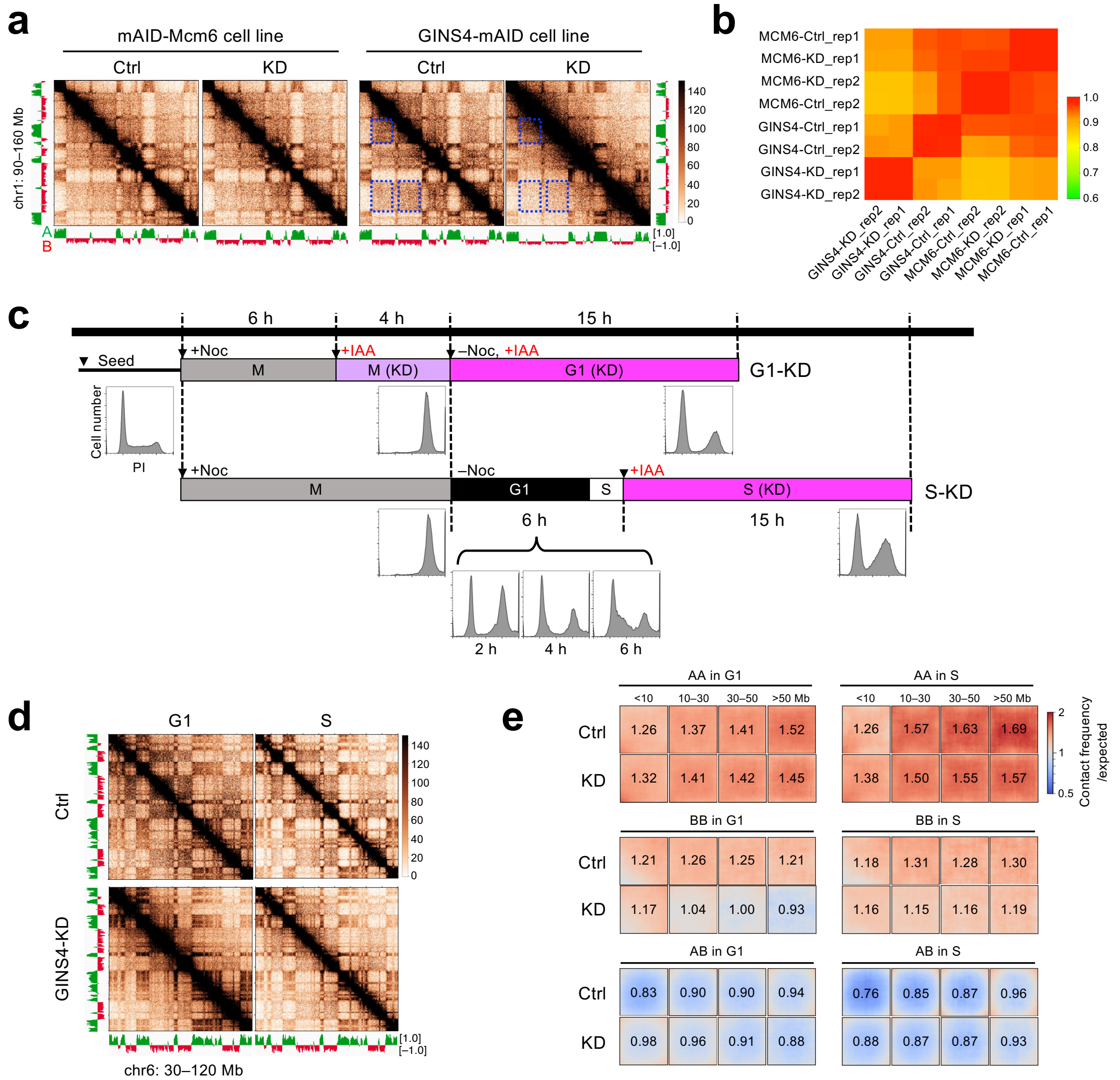

	AA in G1 <10	10–30	30–50	>50 Mb	AA in S <10	10–30	30–50	>50 Mb
Ctrl	1.26	1.37	1.41	1.52	1.26	1.57	1.63	1.69
KD	1.32	1.41	1.42	1.45	1.38	1.50	1.55	1.57

	BB in G1				BB in S			
Ctrl	1.21	1.26	1.25	1.21	1.18	1.31	1.28	1.30
KD	1.17	1.04	1.00	0.93	1.16	1.15	1.16	1.19

	AB in G1				AB in S			
Ctrl	0.83	0.90	0.90	0.94	0.76	0.85	0.87	0.96
KD	0.98	0.96	0.91	0.88	0.88	0.87	0.87	0.93

Supplementary Figure 4. GINS4-KD cells exhibit A/B compartment defects
(a) Hi-C heatmaps of MCM6- and GINS4-depleted cells (KD, 5-Ph-IAA-treated) and control cells (Ctrl, untreated). Chromosome 1 is shown. PC1 eigenvector values, representing A/B compartments, are shown below and beside the Hi-C heatmaps. Blue boxes indicate B-compartment regions with marked loss of chromosome interaction in GINS4-depleted cells. Two independent experiments yielded similar results. **(b)** The stratum-adjusted correlation coefficient (SCC) was calculated using HiCRep tool (*37*) to assess the similarity among Hi-C datasets from MCM6-KD, GINS4-KD and each control sample (two replicates). **(c)** Verification of cell-cycle arrest efficiency during the synchronization process to obtain G1- and S-phase populations. Cells were stained with propidium iodide. Noc, nocodazole; IAA, 5-Ph-IAA. **(d)** Hi-C heatmaps of G1- or S-phase sorted control and GINS4-KD cells. Chromosome 6 is shown. **(e)** Distance-dependent contact frequency was analyzed using Pentad (*38*). The numbers at the center of the plots indicate the average ratio of observed to expected contact frequency.

Supplementary Figure 5

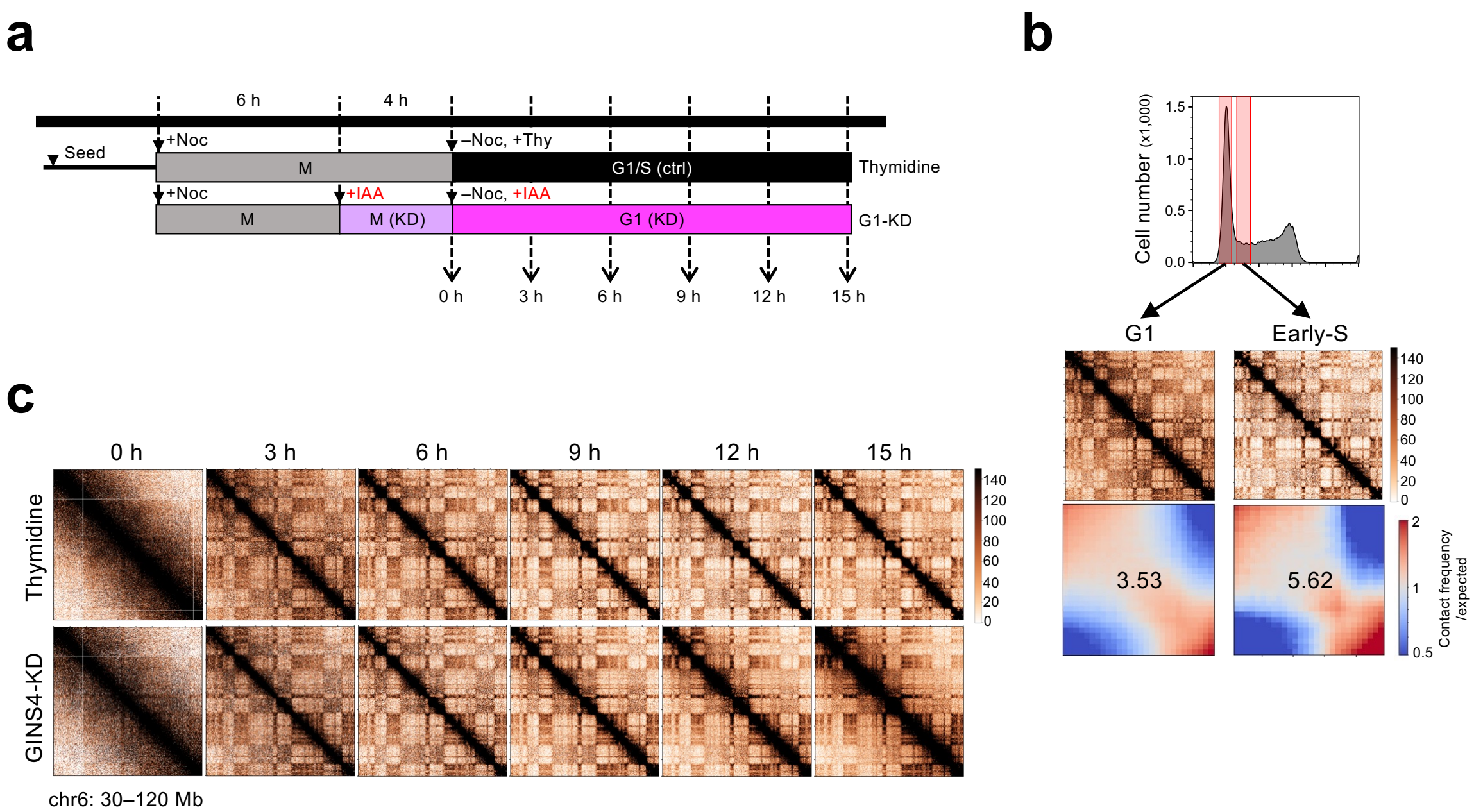

Supplementary Figure 5. Time-course Hi-C analysis following cell-cycle arrest induced by thymidine or 5-Ph-IAA treatment

(a) Scheme of cell cycle arrest and time-course Hi-C sampling. **(b)** Top panel, a FACS profile showing normal G1- and early S-phase cells purified from an asynchronous population. Cell populations highlighted in red were sorted for Hi-C. Cells were stained with propidium iodide. Bottom panel, Hi-C contact heatmaps and saddle plots generated from Hi-C data. **(c)** Hi-C contact heatmaps of thymidine/5-Ph-IAA-treated cells at each time point. Chromosome 6 is shown.

Supplementary Figure 6

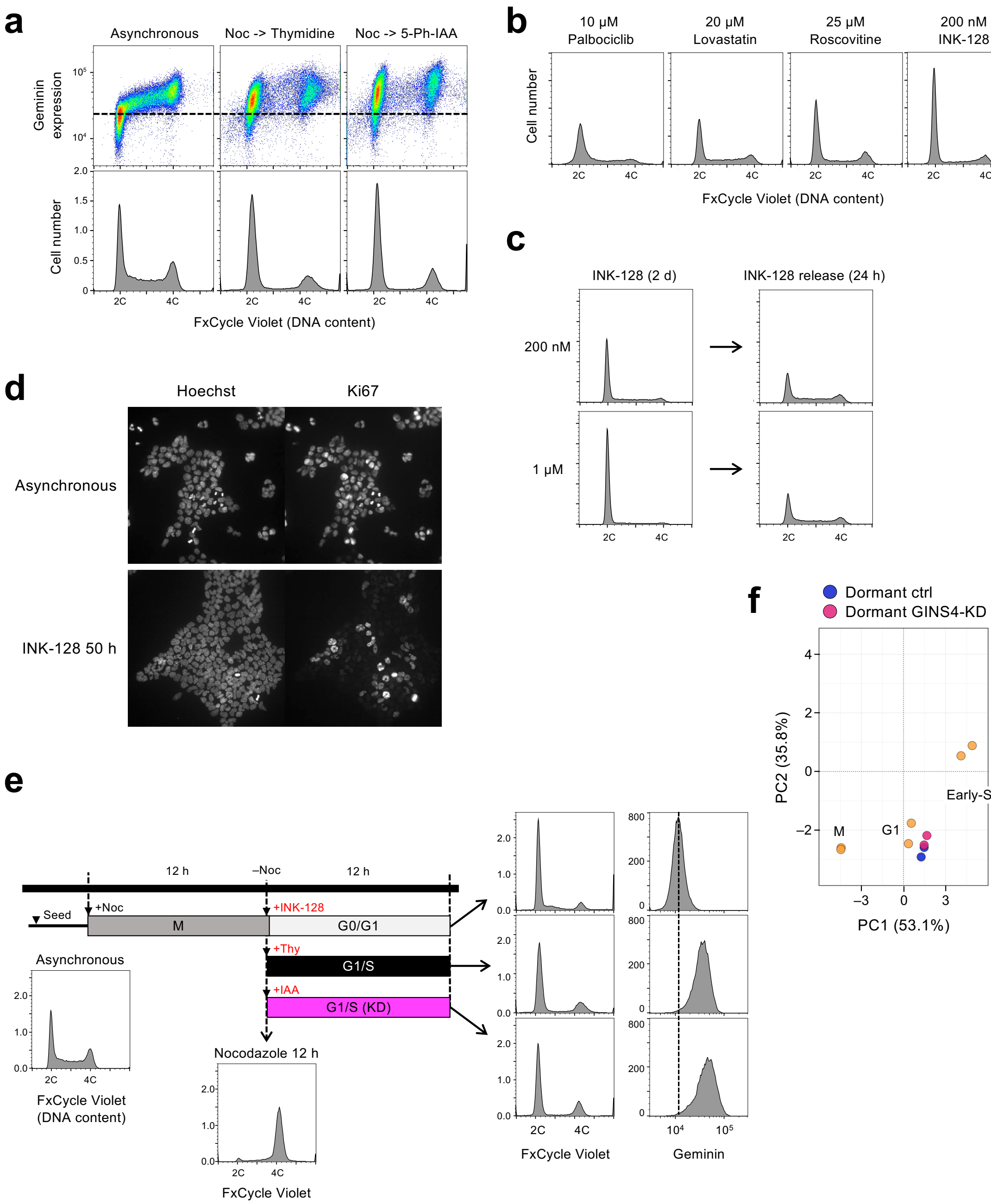

Supplementary Figure 6. Analysis of GINS4 function in non-replicating G0/G1 cells using INK-128

(a) FACS plots of asynchronous cells, thymidine-treated cells after nocodazole release, and 5-Ph-IAA-treated cells after nocodazole release. Both thymidine and 5-Ph-IAA treatments were performed overnight. The upper panels show Geminin expression levels, while the lower panels display cell-cycle profiles based on DNA dye intensity (FxCycle Violet). **(b)** G1 arrest test using various compounds, with INK-128 identified as the most effective reagent for inducing replication arrest in the G1 phase (2N). **(c)** Evaluation of INK-128 treatment at two concentrations, demonstrating that 1 µM more effectively inhibited replication and promoted

robust cell cycle re-entry upon release. **(d)** Immunostaining of Ki67, a cell proliferation marker, to confirm mESC dormancy. Ki67 signals were significantly reduced in most cells following INK-128 treatment. **(e)** Schematic illustrating the process of inducing cell dormancy (G0/G1; INK-128) or G1/S accumulation (thymidine and 5-Ph-IAA). Cell-cycle profiles of asynchronous and drug-treated cells were assessed by FxCycle Violet staining. The right panels show Geminin expression levels determined by immunofluorescence after each drug treatment. Noc, nocodazole; Thy, thymidine; IAA, 5-Ph-IAA. **(f)** PCA plots generated from saddle plot profiles of INK-128-treated cells with or without GINS4 depletion. To compare the cell-cycle status of INK-128-treated cells, Hi-C data from G1-, early S-phase and metaphase cell populations were added as benchmarks, represented by orange plots.

Supplementary Figure 7

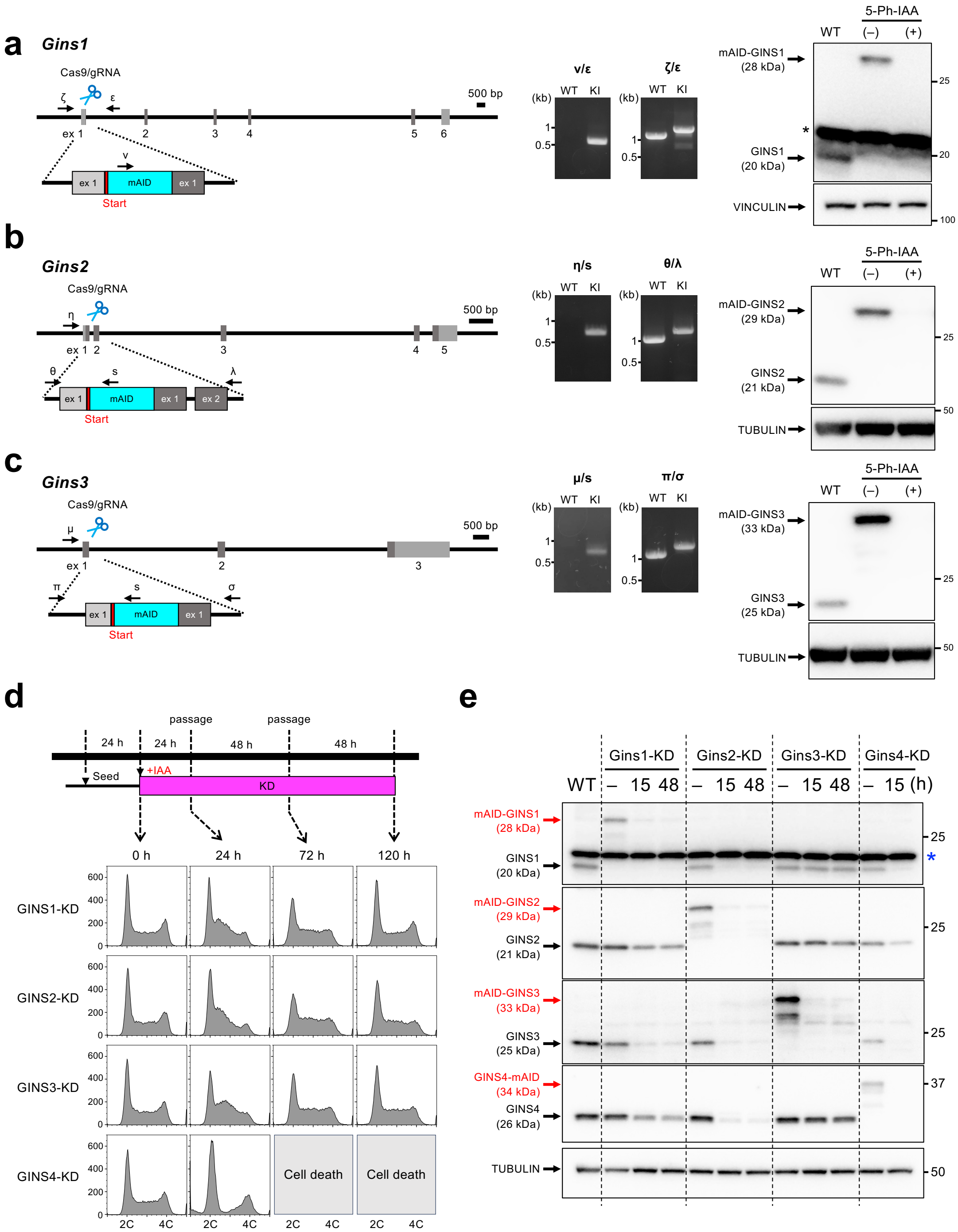

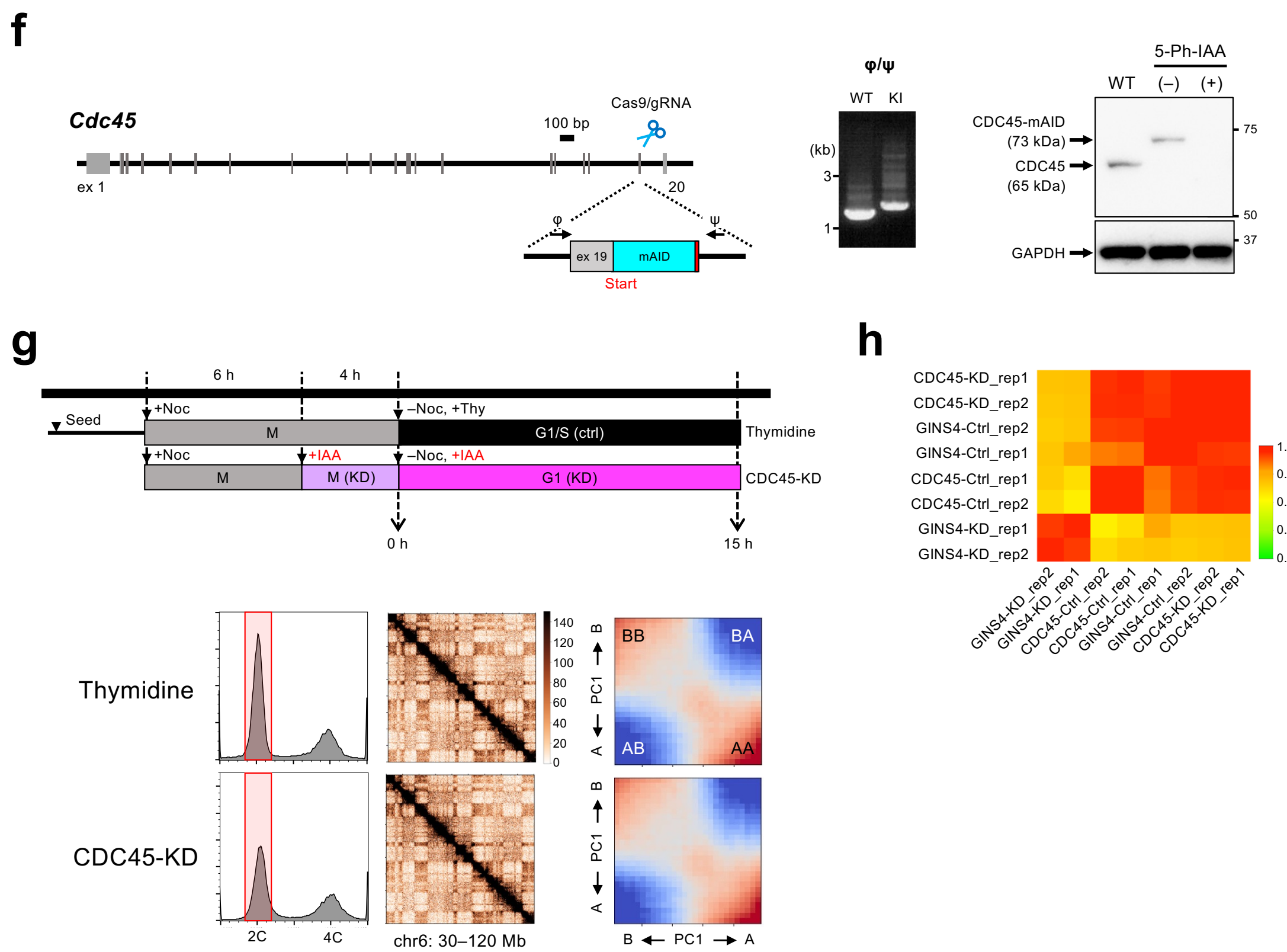

Supplementary Figure 7. Phenotypic analysis of cells with individual depletion of GINS1, GINS2, or GINS3

(a–c) (Left) Knock-in strategy for introducing an mAID tag into the N-terminus of *Gins1, Gins2*, and *Gins3*. CRISPR/Cas9 target sites are indicated by scissors, and genotyping primers are represented by black arrows. Biallelic insertion of mAID tags were confirmed by genotyping PCR (center) and immunoblotting (right). **(d)** Time-course observation of cell-cycle profiles following 5-Ph-IAA treatment in mAID-*Gins1*, mAID-*Gins2*, mAID-*Gins3*, and *Gins4*-mAID cell lines. Unlike GINS4-KD cells, GINS1-, GINS2-, and GINS3-KD cells continued to proliferate after 5-Ph-IAA treatment. Therefore, these cells were passaged every two days in 5-Ph-IAA-containing medium. In contrast, Gins4-KD cells exhibited loss of viability at the 72-hour and 120-hour time points due to cell death. Cells were stained with propidium iodide. **(e)** Immunoblot analysis of each GINS complex component in GINS1-, GINS2-, GINS3-, and GINS4-depleted cells. Cells were collected at 15-hour and 48-hour time points after 5-Ph-IAA treatment, except for GINS4-KD cells, which underwent cell death after prolonged 5-Ph-IAA exposure, as shown in (d). The band positions corresponding to the mAID-tagged GINS components are indicated in red, while the blue asterisk marks an extra non-specific band. **(f)** (Left) Knock-in strategy for introducing an mAID tag into the C-terminus of Cdc45, as in (a). Biallelic insertion was confirmed by genotyping PCR (center) and immunoblotting (right). **(g)** (Top) Scheme of cell-cycle arrest and Hi-C sampling. (Bottom) FACS profiles 15 hours after nocodazole release (left). Cell populations highlighted in red were sorted for Hi-C experiments. Cells were stained with propidium iodide. Hi-C contact heatmaps (center) and saddle plots (right), generated from the Hi-C data, are shown for each sample. **(h)** The stratum-adjusted

correlation coefficient (SCC) was calculated using the HiCRep tool (*37*) to assess similarity among Hi-C datasets from CDC45-KD, GINS4-KD, and each control sample (two replicates).

Supplementary Figure 8

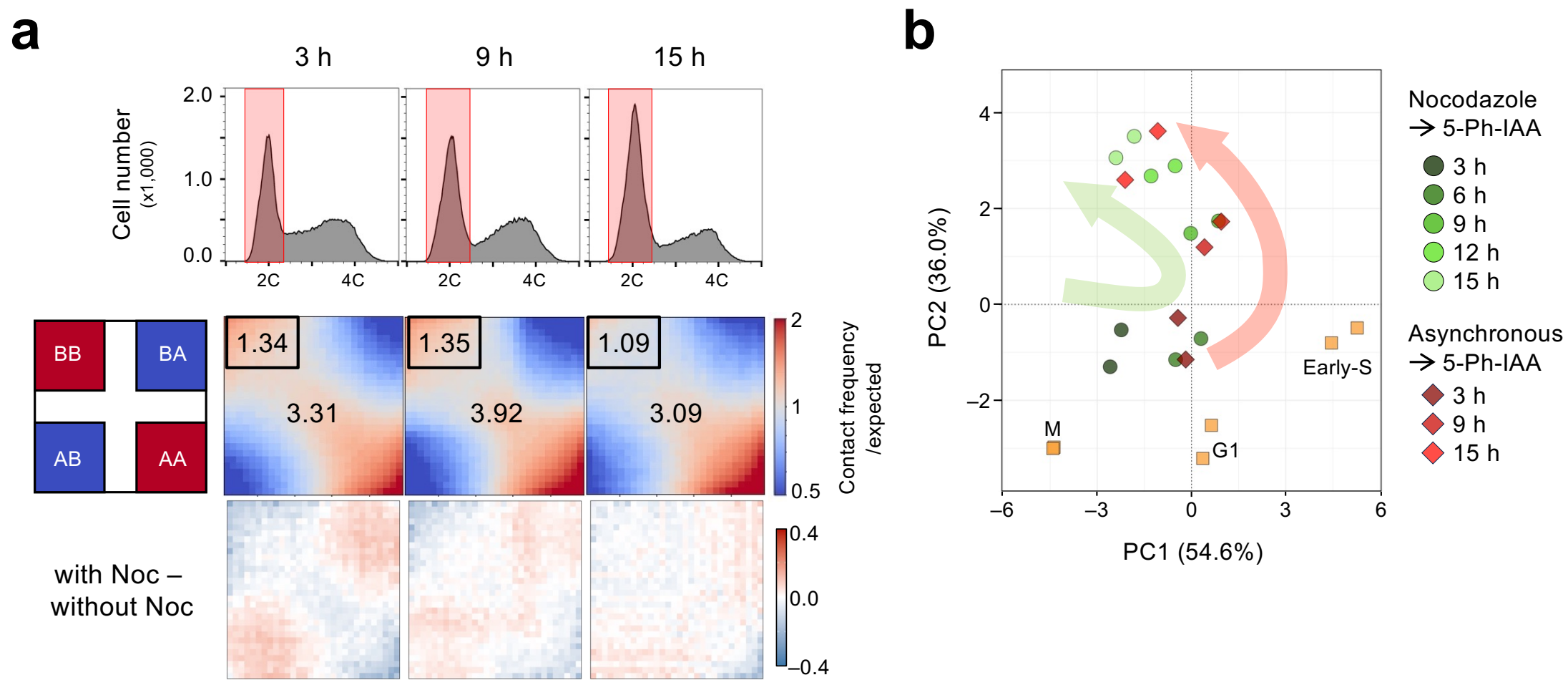

Supplementary Figure 8. Analysis of the GINS4 -KD effect without drug-induced cell-cycle synchronization

(a) Top panels show time-course cell-cycle profiles following 5-Ph-IAA treatment, performed without any prior nocodazole treatment. Red-highlighted cells were sorted for subsequent Hi-C experiments. Cells were stained with propidium iodide. The bottom panels display saddle plots generated from the Hi-C data and subtracted heatmaps (with nocodazole – without nocodazole). The cartoon to the left of the saddle plots indicates the types of compartment contacts at each corner of the saddle plot. Numbers in the top left corners of the saddle plots indicate compartment strength, calculated as the median value of the top 20% BB interactions. Numbers at the center of the heatmap represent compartment strength calculated as the ratio of (AA+BB)/(AB+BA), using the median values of the top 20% AA, BB, and AB interactions. **(b)** PCA plots generated from the saddle plot data of the time-course Hi-C samples shown in (a) and in Fig. 3E (5-Ph-IAA-treated samples). Hi-C data from G1, early S-phase, and metaphase cell populations were included as benchmarks and are represented by orange plots.

Supplementary Figure 9

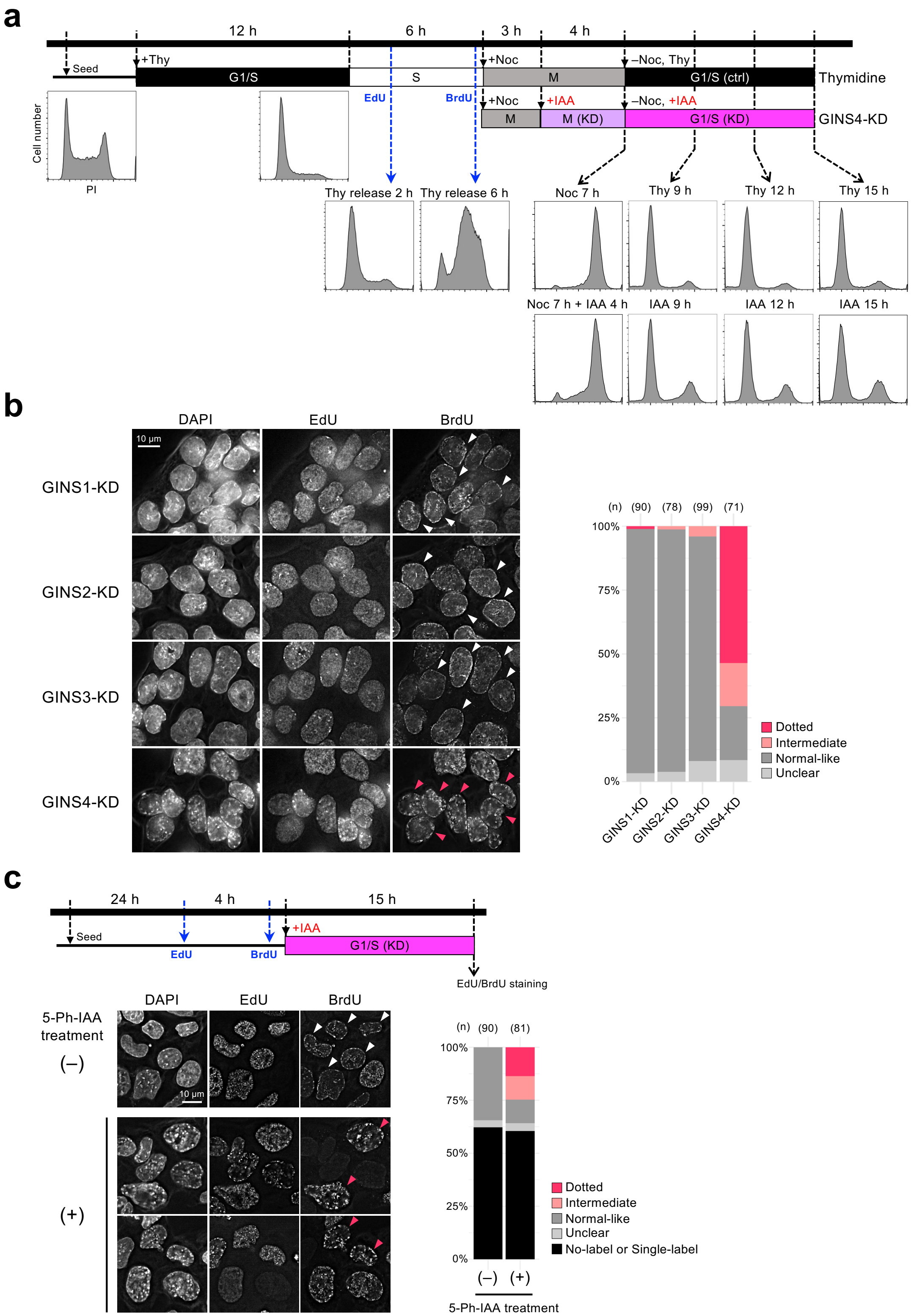

Supplementary Figure 9. Nuclear reorganization process of A and B compartments in the absence of GINS4

(a) Cells cultured for staining were collected at each time point, and cell cycle synchronization efficiency was validated by flow cytometry. Cells were stained with propidium iodide. Noc, nocodazole; Thy, thymidine; IAA, 5-Ph-IAA. **(b)** Representative examples of cells subjected to GINS1, GINS2, GINS3, or GINS4 knockdown after EdU/BrdU pulse labeling, following the experimental schedule described in Figure 4A. The stacked bar graph on the right categorizes cells based on their BrdU signal patterns. White arrowheads indicate normal-like nuclei, while pink arrowheads indicate nuclei with dotted patterns. **(c)** To rule out the possibility that aberrant BrdU foci resulted from multiple drug treatments, EdU/BrdU pulse-labeling was performed before 5-Ph-IAA treatment under asynchronous conditions (see top schematic). Representative images of EdU and BrdU staining, with or without 5-Ph-IAA treatment, are shown on the left. In the stacked bar graph on the right, we categorized BrdU signal patterns exclusively in cells that were double-positive for both EdU and BrdU signals. White arrowheads indicate normal-like nuclei, while pink arrowheads indicate nuclei with dotted patterns.

Supplementary Figure 10

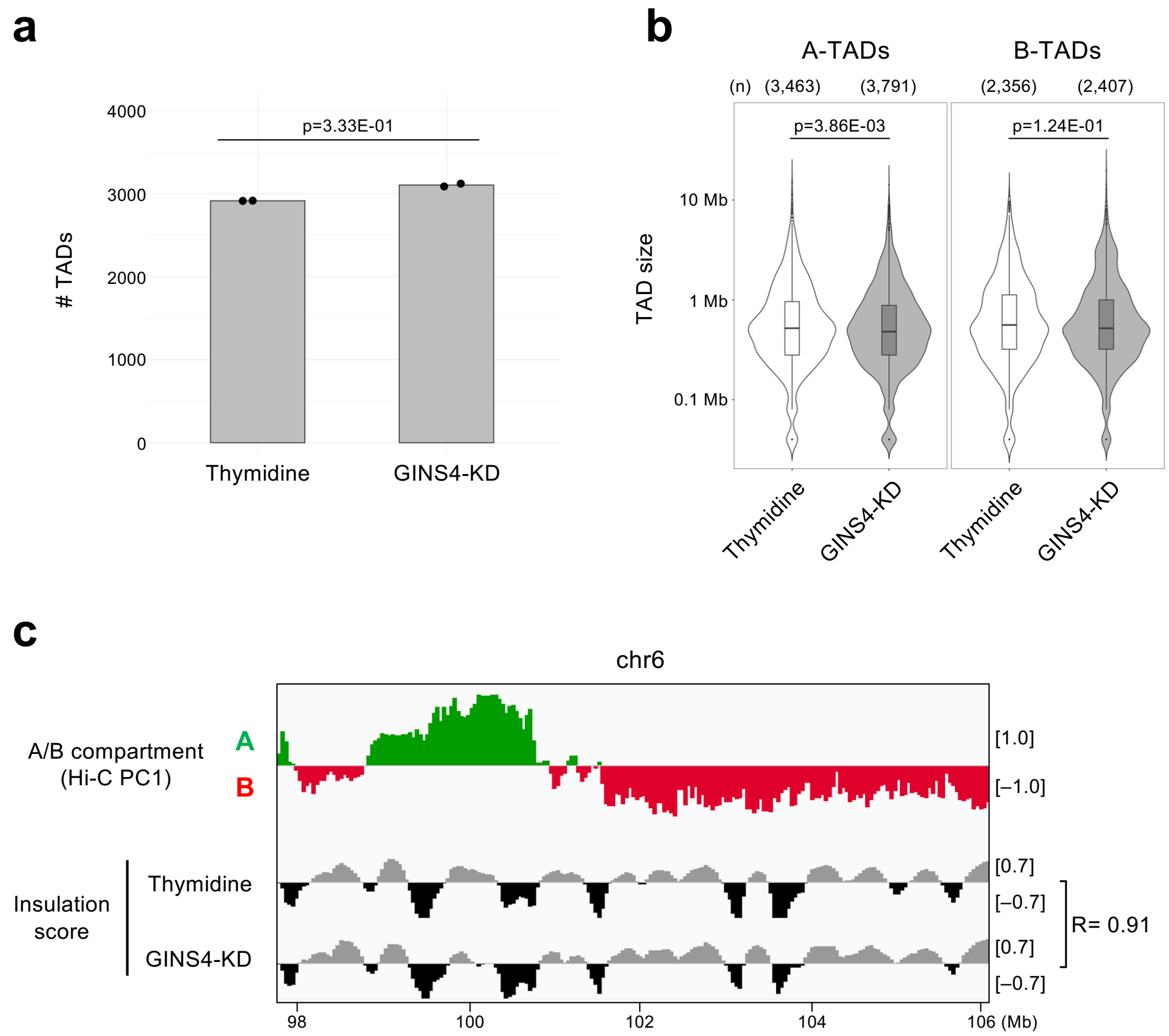

Supplementary Figure 10. The organization of TADs is preserved in GINS4-KD cells
(a) Quantification of the number of TADs. Bar heights indicate the mean values of two biological replicates for each condition, and dots represent individual replicate data. Statistical significance was assessed using the Wilcoxon test. **(b)** Violin plots showing the size distribution of TADs in A (A-TADs) or B compartments (B-TADs). **(c)** An example region on chromosome 6 is shown with the profiles of A/B compartments (Hi-C PC1) and insulation scores used to identify TAD boundaries. The insulation score profiles are highly similar to each other, exhibiting a high Pearson correlation coefficient.

Supplementary Figure 11

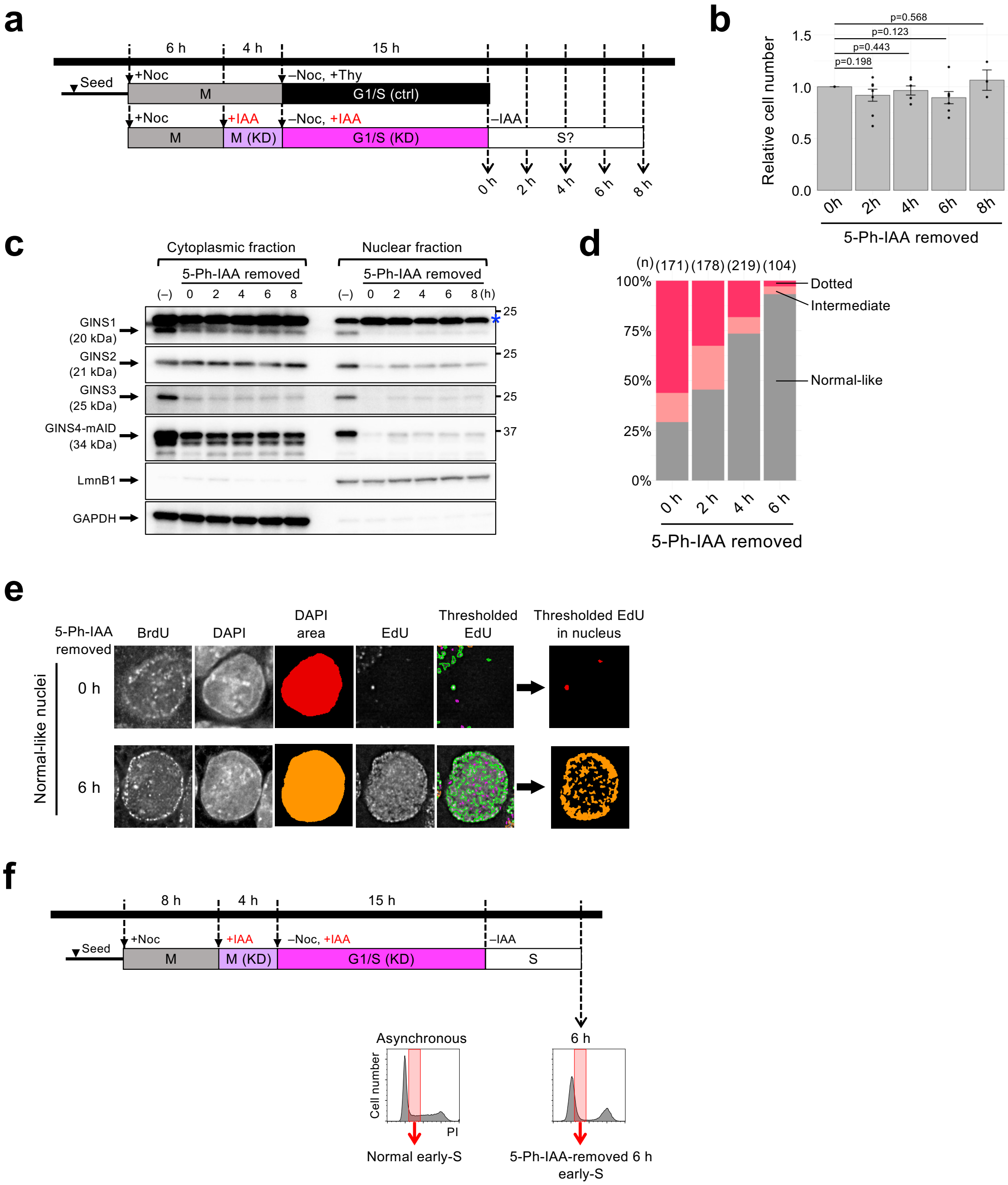

Supplementary Figure 11. Restoration of DNA replication and A/B compartment structure upon GINS4 re-synthesis

(a) Schematic illustrating GINS4 restoration and the time-course sampling to monitor cell-cycle progression after 5-Ph-IAA removal. Noc, nocodazole; Thy, thymidine; IAA, 5-Ph-IAA. **(b)** Relative cell number was calculated by setting 0 h time point (immediately after 5-Ph-IAA removal) as 1.0. Dead cells were washed off before counting, and therefore only surviving cells were counted. Each time point represents data from more than three replicates. P values were calculated using two-sided one-sample t-tests against 1.0. **(c)** Immunoblot analysis of GINS1, GINS2, and GINS3 proteins in *Gins4*-mAID cells after 15 h of 5-Ph-IAA treatment (0 h) and

during recovery (2–8 h post 5-Ph-IAA removal). Cell fractionation was performed to assess the recovery of the GINS complex in both nuclear and cytoplasmic fractions. LmnB1 and GAPDH served as loading controls for the nuclear and cytoplasmic fractions, respectively. **(d)** Alteration in the spatial patterns of BrdU incorporation after 5-Ph-IAA removal. Unlike Fig. 4D, correctly-labeled nuclei were not distinguishable due to the absence of an EdU label in early-replicating DNA. Therefore, only nuclei with clear BrdU signal patterns were selected and classified into three categories: dotted, intermediate, and normal-like. **(e)** Examples of EdU signal quantification in individual nuclei using CellProfiler. Note that the threshold settings for EdU signal detection were not perfect, as seen in the nucleus at 6 h after 5-Ph-IAA removal. **(f)** Schematic of the Hi-C experiment 6 hours after 5-Ph-IAA removal. The ‘5-Ph-IAA-removed 6 h early-S’ population was sorted using the same gating strategy as for the ‘normal early-S’ sorted from asynchronous cells. Cells were stained with propidium iodide.

Supplementary Figure 12

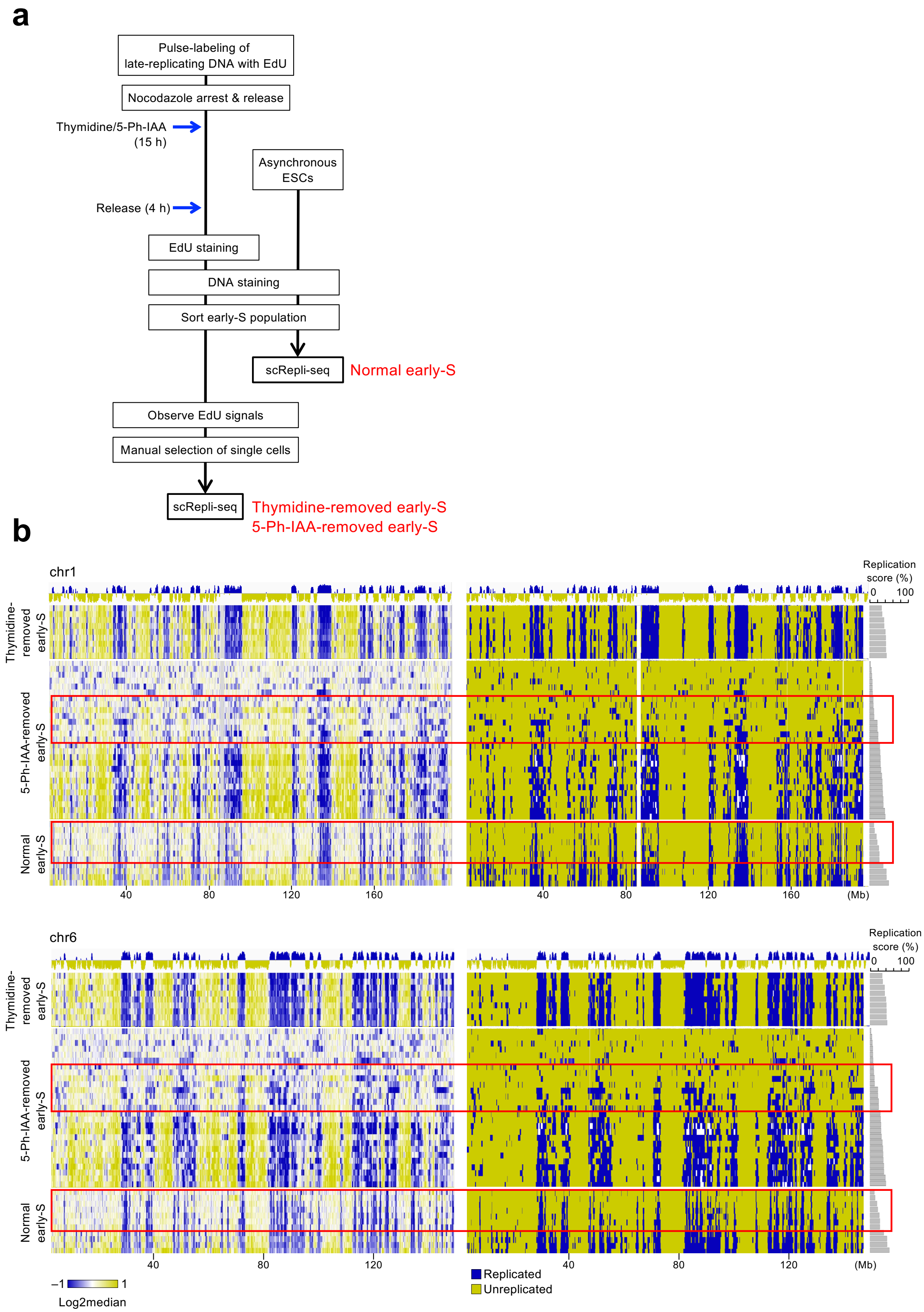

Supplementary Figure 12. Single-cell Repli-seq of GINS4-restored cells in early S-phase
(a) Experimental workflow for collecting EdU-labeled GINS4-restored cells in early S-phase for scRepli-seq. Cells treated with thymidine or 5-Ph-IAA were manually collected using a mouth pipette after microscopic observation of EdU signals. In contrast, the 'normal early-S' cells without EdU labeling were directly sorted for scRepli-seq to represent normal RT in early S-phase. **(b)** Log2-median scRepli-seq profiles (left) and binarized scRepli-seq profiles (right) of three types of 'early S-phase' cells collected as described in (a). Chromosomes 1 and 6 are shown as examples. The top panels for each chromosome display representative RT peaks obtained from population-based BrdU-IP Repli-seq performed on *Gins4*-mAID cells without 5-Ph-IAA treatment (see Figure 2b). Cells are ordered by percentage replication scores. Red boxes indicate specific cells with replication scores ranging from 10% to 25%.
