# Supplementary material for: Nuclear compartmentalization at the G1/S transition plays a key role in DNA replication control
Source: Nat Commun. 2026 Aug 10;17:6961. doi: 10.1038/s41467-026-75264-6 (PMC13458202; doi:10.1038/s41467-026-75264-6)
Supplement: Supplementary file 2 [file 41467_2026_75264_MOESM2_ESM.pdf]

Description of Additional Supplementary Files

File Name: Supplementary Data 1
Description: Primer list

File Name: Supplementary Data 2
Description: Plasmid list

File Name: Supplementary Data 3
Description: gRNA list

File Name: Supplementary Data 4
Description: List of sequencing experiments
