# Supplementary material for: Nuclear compartmentalization at the G1/S transition plays a key role in DNA replication control
Source: Nat Commun. 2026 Aug 10;17:6961. doi: 10.1038/s41467-026-75264-6 (PMC13458202; doi:10.1038/s41467-026-75264-6)
Supplement: Supplementary file 3 [file 41467_2026_75264_MOESM4_ESM.pdf]

nature portfolio

Corresponding author(s): Ichiro Hiratani and Asami Oji

Last updated by author(s): Jun 2, 2026

Reporting Summary

Nature Portfolio wishes to improve the reproducibility of the work that we publish. This form provides structure for consistency and transparency in reporting. For further information on Nature Portfolio policies, see our Editorial Policies and the Editorial Policy Checklist.

Statistics

For all statistical analyses, confirm that the following items are present in the figure legend, table legend, main text, or Methods section.

n/a	Confirmed	
☐	☒	The exact sample size (*n*) for each experimental group/condition, given as a discrete number and unit of measurement
☐	☒	A statement on whether measurements were taken from distinct samples or whether the same sample was measured repeatedly
☐	☒	The statistical test(s) used AND whether they are one- or two-sided<br>*Only common tests should be described solely by name; describe more complex techniques in the Methods section.*
☒	☐	A description of all covariates tested
☐	☒	A description of any assumptions or corrections, such as tests of normality and adjustment for multiple comparisons
☐	☒	A full description of the statistical parameters including central tendency (e.g. means) or other basic estimates (e.g. regression coefficient) AND variation (e.g. standard deviation) or associated estimates of uncertainty (e.g. confidence intervals)
☐	☒	For null hypothesis testing, the test statistic (e.g. *F*, *t*, *r*) with confidence intervals, effect sizes, degrees of freedom and *P* value noted<br>*Give P values as exact values whenever suitable.*
☒	☐	For Bayesian analysis, information on the choice of priors and Markov chain Monte Carlo settings
☒	☐	For hierarchical and complex designs, identification of the appropriate level for tests and full reporting of outcomes
☐	☒	Estimates of effect sizes (e.g. Cohen's *d*, Pearson's *r*), indicating how they were calculated

Our web collection on statistics for biologists contains articles on many of the points above.

Software and code

Policy information about availability of computer code

Data collection	Sony SH800 software version 2.1 Sony MA900 software version 3.2 were used for flow cytometry analysis. Images were colleted by DeltaVision Olympus IX71 inverted microscope and a standard SoftWoRx acquisition software version 6.5.2.
Data analysis	Analytical softwares used for Hi-C are available under Github: https://github.com/4dn-dcic/docker-4dn-hic, https://github.com/dewitlab/GENOVA, https://github.com/magnitov/pentad. Analytical softwares for Repli-seq (after BrdU-IP) and scRepli-seq are available under Github: https://github.com/kuzobuta/scRepliseq-Pipeline. The flow cytometry results were analyzed using FlowJo™ v10.9.0 Software (BD Life Sciences). Imaging data were processed and analyzed using Fiji (Schindelin J et al. 2012) and CellProfiler v4.2.5 (Stirling DR et al. 2021).

For manuscripts utilizing custom algorithms or software that are central to the research but not yet described in published literature, software must be made available to editors and reviewers. We strongly encourage code deposition in a community repository (e.g. GitHub). See the Nature Portfolio guidelines for submitting code & software for further information.

Data

Policy information about availability of data

All manuscripts must include a data availability statement. This statement should provide the following information, where applicable:

- Accession codes, unique identifiers, or web links for publicly available datasets
- A description of any restrictions on data availability
- For clinical datasets or third party data, please ensure that the statement adheres to our policy

All data of this study are stored at the corresponding author and available request.
All data has been deposited in the Gene Expression Omnibus under the accessions GSE245938. mm9 reference genome (UCSC) was used.
Reviewer token is "ozadayaytlkzpyf".

Research involving human participants, their data, or biological material

Policy information about studies with human participants or human data. See also policy information about sex, gender (identity/presentation), and sexual orientation and race, ethnicity and racism.

Reporting on sex and gender	Human research is not involved in this study.
Reporting on race, ethnicity, or other socially relevant groupings	Human research is not involved in this study.
Population characteristics	Human research is not involved in this study.
Recruitment	Human research is not involved in this study.
Ethics oversight	Human research is not involved in this study.

Note that full information on the approval of the study protocol must also be provided in the manuscript.

Field-specific reporting

Please select the one below that is the best fit for your research. If you are not sure, read the appropriate sections before making your selection.

☒ Life sciences ☐ Behavioural & social sciences ☐ Ecological, evolutionary & environmental sciences

For a reference copy of the document with all sections, see nature.com/documents/nr-reporting-summary-flat.pdf

Life sciences study design

All studies must disclose on these points even when the disclosure is negative.

Sample size	No sample-size calculation was performed. The sample size was chosen based on previous experience and standards in the field.
Data exclusions	For scRepli-seq analysis, we treated the data as described in Miura et al., Nature Protocols 2020. From a total 62 single-cell data sets of ESCs, we excluded 8 that did not pass the 'MAD score' QC, 1 that showed problematic replication timing (RT) data distribution by the manhattan distance, as well as 7 with chromosome abnormalities and used the remaining 47 data sets for downstream scRepli-seq analysis.
Replication	All experiments were replicated at least twice, and in all cases, data sets exhibited high correlation among the replicates, assuring data reproducibility. Biological replicates represented by cells from different passages and different collected date.
Randomization	Randomization is not relevant to this study because no comparisons between experimental groups were made.
Blinding	Blinding was not applied because experimental groups were defined by treatment or sorting conditions, and most analyses were performed using predefined FACS gates, automated image quantification, or genome-wide sequencing pipelines with fixed parameters. For image-based pattern classification, categories and criteria were defined before quantification and applied consistently across samples.

Reporting for specific materials, systems and methods

We require information from authors about some types of materials, experimental systems and methods used in many studies. Here, indicate whether each material, system or method listed is relevant to your study. If you are not sure if a list item applies to your research, read the appropriate section before selecting a response.

Materials & experimental systems

n/a	Involved in the study
☐	☒ Antibodies
☐	☒ Eukaryotic cell lines
☒	☐ Palaeontology and archaeology
☒	☐ Animals and other organisms
☒	☐ Clinical data
☒	☐ Dual use research of concern
☒	☐ Plants

Methods

n/a	Involved in the study
☒	☐ ChIP-seq
☐	☒ Flow cytometry
☒	☐ MRI-based neuroimaging

Antibodies

Antibodies used

anti-OsTIR1 (1:2000, a gift from Dr. Masato Kanemaki)
anti-MCM6 (1:5000, SantaCruz, sc-393618)
anti-ORC1 (1:1000, Cell Signaling, #4731)
anti-GINS4 [SLD5] (1:500, GeneTex, GTX119779)
anti-GINS1 [PSF1] (1:300, Abcam, ab181112)
anti-GINS2 [PSF2] (1:1000, Proteintech, 16247-1-AP)
anti-GINS3 (1:500, Proteintech, 15651-1-AP)
anti-CDC45(1:1000, Cell Signaling, #3673)
anti-Geminin (1:400, Abcam, ab175799)
anti-GAPDH (1:8000, Abcam, ab8245)
anti-alpha TUBULIN (1:10000, Abcam, ab7291)
anti-Vinculin (1:500000, Sigma, V9131)
anti-BrdU antibody (0.5 µg, BD Biosciences Pharmingen, 555627 for BrdU-IP, 1:100, Abcam, ab8039 for staining)

Validation

Commercially available antibodies are accompanied by validation datasets provided by the suppliers. In addition, gifted antibodies have been previously validated in published studies.
anti-OsTIR1 (validated in the previous study: Yesbolatova et al., Nature Communications. 2020)
anti-MCM6 (https://www.scbt.com/p/mcm6-antibody-h-8)
anti-ORC1 (https://www.cellsignal.jp/products/primary-antibodies/orc1-7a7-rat-mab/4731)
anti-GINS4 [SLD5] (https://search.cosmobio.co.jp/view/p_view.asp?PrimaryKeyValue=3267864&ServerKey=&selPrice=1)
anti-GINS1 [PSF1] (https://www.abcam.co.jp/products/primary-antibodies/psf1-antibody-epr13359-ab181112.html)
anti-GINS2 [PSF2] (https://www.ptglab.com/products/GINS2-Antibody-16247-1-AP.htm)
anti-GINS3 (https://www.ptglab.com/products/GINS3-Antibody-15651-1-AP.htm)
anti-CDC45 (https://www.cellsignal.com/products/primary-antibodies/cdc45-antibody/3673?srsltid=AfmBOoq0i7j_4h3RamIVQM0duKI9R7A1Pb3Uk3bNUChes9SjvIKBsK3n)
anti-Geminin (https://www.abcam.co.jp/products/primary-antibodies/geminin-antibody-ab175799.html)
anti-GAPDH (https://www.abcam.co.jp/products/primary-antibodies/gapdh-antibody-6c5-loading-control-ab8245.html)
anti-alpha TUBULIN (https://www.abcam.co.jp/products/primary-antibodies/alpha-tubulin-antibody-dm1a-loading-control-ab7291.html)
anti-Vinculin (https://www.sigmaaldrich.com/JP/en/product/sigma/v9131)
anti-BrdU antibody for BrdU-IP (validated in the previous study: Takahashi et al., Nature Genetics 2019)
anti-BrdU antibody for staining (https://www.abcam.co.jp/products/primary-antibodies/brdu-antibody-mobu-1-ab8039.html)

Eukaryotic cell lines

Policy information about cell lines and Sex and Gender in Research

Cell line source(s)	mouse embryonic stem cells, mESCs (generated by Dr. Yoshitaka Fujihara, Fujihara et al., Transgenic Res 2013)
Authentication	Karyotypes were verified using an NGS technology.
Mycoplasma contamination	The cell line tested negative for mycoplasma contamination.
Commonly misidentified lines (See ICLAC register)	No commonly misidentified cell lines were used.

Plants

Seed stocks

No plants were used in this study.

Novel plant genotypes

No plants were used in this study.

Authentication

No plants were used in this study.

Flow Cytometry

Plots

Confirm that:

☒ The axis labels state the marker and fluorochrome used (e.g. CD4-FITC).

☒ The axis scales are clearly visible. Include numbers along axes only for bottom left plot of group (a 'group' is an analysis of identical markers).

☒ All plots are contour plots with outliers or pseudocolor plots.

☒ A numerical value for number of cells or percentage (with statistics) is provided.

Methodology

Sample preparation

Repli-seq and observation of cell cycle profiles: Cells were fixed with 75% ethanol, and then stained with propidium iodide (PI) or FxCycle™ Violet Stain (Invitrogen) to assess the DNA content of the cell as previously described (Takahashi et al., Nature Genetics 2019). DNA content was analyzed using Sony SH800 and Sony MA900 cell sorter.
Hi-C: Cells were fixed with 1% formaldehyde, and then permeabilized by 0.1% saponin, then stained with PI to assess the DNA content of the cell as previously described (Bonev et al., Cell 2017).

Instrument

Sony SH800 Cell sorter and Sony MA900 Cell sorter (SONY).

Software

The data was collected by Cell Sorter Software version 2.1 (SH800) and Cell Sorter Software version 3.2 (MA900). Cell cycle profiles were generated using FlowJo™ v10.9.0 Software.

Cell population abundance

100,000-2,000,000 cells were acquired for each sample. Cells were first examined by forward and back scatter (FSC and BSC), and the major population (70–90%) was purified before analyzing each fluorescence.

Gating strategy

An FSC/BSC gate was used for gating the population of cells to exclude cell debris. Then, a PI gate was used to exclude doublet cells. The gates for sorting the G1, G2/M or desired S-phase fractions were defined on the PI histogram. Cells were sorted with the purity mode of the SH800 or MA900 Cell Sorter. For scRepli-seq, cells were sorted using the single-cell mode.

☒ Tick this box to confirm that a figure exemplifying the gating strategy is provided in the Supplementary Information.
